# Targeting papillomavirus infections: high-throughput screening reveals an effective inhibitor of cutaneous β-HPV types

**DOI:** 10.1128/jvi.00918-25

**Published:** 2025-07-08

**Authors:** Ruslan Ibragimov, Sofiya Babok, Elina Lototskaja-Perepelenko, Larisa Ivanova, Nika Mikhailava, Lidiia Zaikina, Marko Piirsoo, Alla Piirsoo

**Affiliations:** 1 Institute of Technology, University of Tartu 124633, Tartu, Estonia; 2 Institute of Chemistry, University of Tartu 428488 , Tartu, Estonia; 3 Institute of Bioengineering, University of Tartu 37546, Tartu, Estonia; College of Agriculture & Life Sciences, University of Arizona, Tucson, Arizona, USA

**Keywords:** human papillomavirus, high-throughput screening, replication, inhibition, E2 protein

## Abstract

Cutaneous and mucosal human papillomaviruses (HPVs) are implicated in the development of various cancers. To date, there are no HPV-specific antivirals, and vaccines designed for mucosal HPV types are ineffective against cutaneous HPV variants and ongoing infections. These facts, along with the increasing prevalence of specific types of HPV-positive cancers, underscore the unmet need for HPV antiviral drugs. In this study, we performed a high-throughput screening using the National Cancer Institute Diversity Set VI library of small-molecule compounds and identified 5,7-dimethoxy-2-pyridin-3-ylchromen-4-one (NSC51349) as a potent inhibitor of oncogenic cutaneous HPV5 replication in U2OS cells. Further analysis showed that NSC51349 specifically inhibits replication of the HPV5 genome in human primary keratinocytes (IC50 ≈ 4 µM) without detrimental effects on cell cycle, viability, or differentiation. In addition, this compound efficiently inhibits replication of the other cutaneous HPV types 8 and 38, and *Macaca fascicularis* PV type 1 genomes. The mechanism of NSC51349-mediated inhibition involves the suppression of the viral protein E2 transcriptional activity. Molecular docking predicted that NSC51349 interacts with the HPV5 E2 protein by binding to the threonine 202/proline 203 and threonine 473/glutamate 474 amino acid residues, located at the junction of the transactivation domain and hinge region, and the DNA-binding domain, respectively. Our study suggests that NSC51349 is a promising lead compound for further development of cutaneous HPV antiviral drugs.

**IMPORTANCE:**

Human papillomaviruses (HPVs) are linked to various cancers of the skin and mucous membranes. While vaccines exist for some mucosal HPV types, they are ineffective against skin-infecting variants and cannot treat existing infections. This highlights the urgent need for HPV-specific antiviral drugs. In this study, we identified a promising lead compound, 5,7-dimethoxy-2-pyridin-3-ylchromen-4-one (NSC51349), through high-throughput screening of the Diversity Set VI library of small molecules. NSC51349 inhibits the replication of HPV5, a cancer-associated skin virus, in human cells without affecting cell viability, growth, or differentiation. It also inhibits the replication of other cutaneous HPV types, including HPV8 and HPV38, further supporting its broad potential. NSC51349 targets the viral protein E2, which is crucial for HPV replication. By binding to specific regions of E2, it interferes with its transcriptional activity, halting viral replication and the expression of viral oncogenes. This study introduces NSC51349 as a strong candidate for antiviral drug development.

## INTRODUCTION

Human papillomaviruses (HPVs) are small non-enveloped double-stranded DNA viruses that infect mucosal or cutaneous epithelial keratinocytes. Almost 450 different types of HPVs subdivided into five genera (mostly mucosal α-, and cutaneous β-, γ-, µ-, and ŋ-types) have been described to date (1). Although the majority of HPV infections are transient, persistent infection with high-risk (HR) HPVs may induce cellular transformation and cancer. A small group of oncogenic HPVs (up to 17 types) is responsible for approximately 4.5% of all cancers and approximately 31% of infection-caused cancers worldwide (2, 3).

Pathologies associated with mucosal α-HPVs (e.g., α-HPV types 16, 18, and 31) include cervical cancer and its precancerous lesions, other anogenital cancers and warts, and specific types of head-and-neck cancers, such as recurrent respiratory papillomatosis (4). Cutaneous HPV infections are generally asymptomatic. However, under certain conditions, pro-oncogenic HPVs (e.g., 5, 8, or 38) can lead to various skin lesions, such as macules and warts, and, in combination with UV light and other co-carcinogens, can contribute to the neoplastic transformation of keratinocytes and the development of non-melanoma skin cancer, particularly in individuals with epidermodysplasia verruciformis, WHIM syndrome, or immunocompromised conditions, such as HIV infection, organ transplant recipients (OTRs), or cancer patients undergoing treatment (5–9).

Initial steps of HPV infection, common to both types of epithelium, involve viral particle entry, release of the HPV genome into the nucleus, and its replication as an extrachromosomal plasmid. Only two viral proteins, the helicase E1 and transcription factor E2, are required for viral genome replication. Differences between cutaneous and mucosal HPVs emerge during persistent infection, essential for HPV-caused pathologies. In mucosal epithelium, oncogenic HPV DNA often integrates into the host cell genome (10, 11). This integration is accompanied by extensive upregulation of viral oncogenes E6 and E7 expression. E6/E7 activities disrupt normal cellular processes such as the cell cycle, differentiation, senescence, and apoptosis, being prerequisites for cellular transformation and cancer (12). In such cases, targeting HPV replication becomes ineffective, and efforts should focus on inhibiting E6/E7 activities. By contrast, cutaneous viral DNA rarely integrates into the host cell genome (10, 11). Instead, it persists as a multicopy plasmid in the nucleus, replicating in concert with host cell DNA. This feature opens the possibility of targeting viral replication for developing antiviral drugs.

To date, there are no specific antivirals against HPV infections (13, 14). A number of natural and synthetic compounds have been explored to combat HPV infections (14). However, these compounds either target host cell proteins or provide cytotoxic, pro-apoptotic, or immunomodulating effects. Also, vaccines targeting up to nine types of mucosal α-HPVs (HR types 16, 18, 31, 33, 45, 52, and 58, and low-risk types 6 and 11) have been developed and are widely used, but they are inefficient against existing infections and cutaneous HPV types and cannot be used for OTRs (15–17). These facts, along with the growing incidence of HPV-dependent cancers among the vaccine-ineligible population and the escalating number of organ transplantations globally, clearly indicate an unmet medical need for drugs that prevent pathological changes associated with mucosal and cutaneous HPV infections.

Several inhibitors of the mucosal HPV18 have been identified in high-throughput screening (HTS) using the National Cancer Institute (NCI) Diversity Set IV library of small-molecule compounds (18). In this study, we performed HTS on the NCI Diversity Set VI library, which comprises 1,584 chemicals, to identify inhibitors for HPV type 5—an HR virus that infects cutaneous epithelium and does not integrate into the host cell genome. Two compounds, NSC4263 or 5-Nitro-1,10-phenanthroline and NSC51349 or 5,7-dimethoxy-2-pyridin-3-ylchromen-4-one, were identified in the initial screen and analyzed further to evaluate their potential as candidates for the development of antiviral drugs against cutaneous HPV infections.

## RESULTS

### The identified HPV5 inhibitors reveal different specificity toward certain HPV types

**Fig 1:**
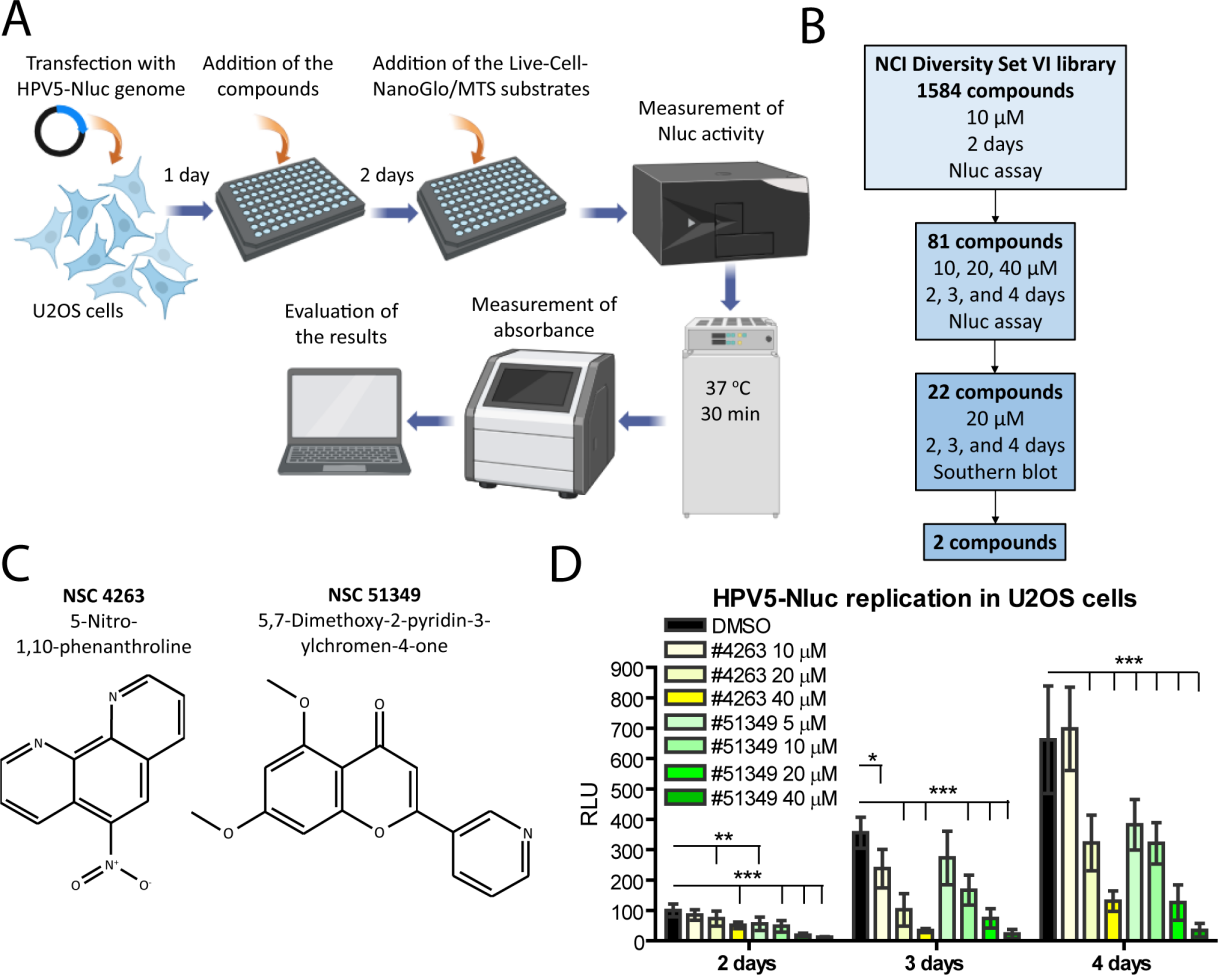
Identified inhibitors of the HPV5-Nluc genome replication. (A, B) Workflow of the HTS performed to identify inhibitors of HPV5-Nluc replication. The concentrations of the chemicals used, incubation times, and biological assays are specified for each round of testing. (A) The image was generated using BioRender software (license no. https://BioRender.com/a24a592). (C) Molecular structure of 5-Nitro-1,10-phenanthroline (NSC4263) and 5,7-dimethoxy-2-pyridin-3-ylchromen-4-one (NSC51349). (D) Replication of the HPV5-Nluc genome was analyzed using a luciferase assay at the indicated time points. The next day after transfection, cells were treated in quadruplicate with different concentrations of the compounds or with the diluent DMSO as a control. Nluc activity was normalized to total protein concentrations or AP activity, and the normalized Nluc activity in control cells treated with DMSO, 2 days post-transfection, was set to 100%. All other data are calculated relative to the control sample. Data are presented as the average percentage of at least three independent experiments ± SD; *P < 0.05, **P < 0.01, ***P < 0.001; RLU, relative luminescence units.

By exploiting the U2OS cell line and high-efficiency transfection of HPV genomes, we have developed a robust, cost-effective cellular system to measure the replication efficiency of different α- and β-HPV types (19, 20). This system has proven useful in HTS of α-HPV18 inhibitors, providing a feasible method for identifying compounds that interfere with viral replication (18). To identify inhibitors of β-HPV5 replication, we performed HTS using the NCI Diversity Set VI library of small-molecule compounds (1,584 chemicals), a modified HPV5 genome expressing Nluc and U2OS cells. The HTS was conducted according to the scheme depicted in Fig. 1A and B. The results of the initial HTS, showing the average Nluc activity values from triplicates, normalized to the number of viable cells in each well (± SD), are presented in Table S1. The data are calculated relative to the normalized Nluc activity in dimethyl sulfoxide (DMSO)-treated control cells in each plate, which was set to 100%. Further validation of the inhibitor candidates resulted in the identification of two compounds, NSC4263 or 5-Nitro-1,10-phenanthroline and NSC51349 or 5,7-dimethoxy-2-pyridin-3-ylchromen-4-one (Fig. 1C). These compounds were selected for further investigation based on their ability to inhibit HPV5-Nluc in a concentration-dependent manner in the Nluc assay (Fig. 1D). In U2OS cells, the IC_50_ values were 14 ± 1.6 µM and 8 ± 1.1 µM for NSC4263 and NSC51349 compounds, respectively.

**Fig 2:**
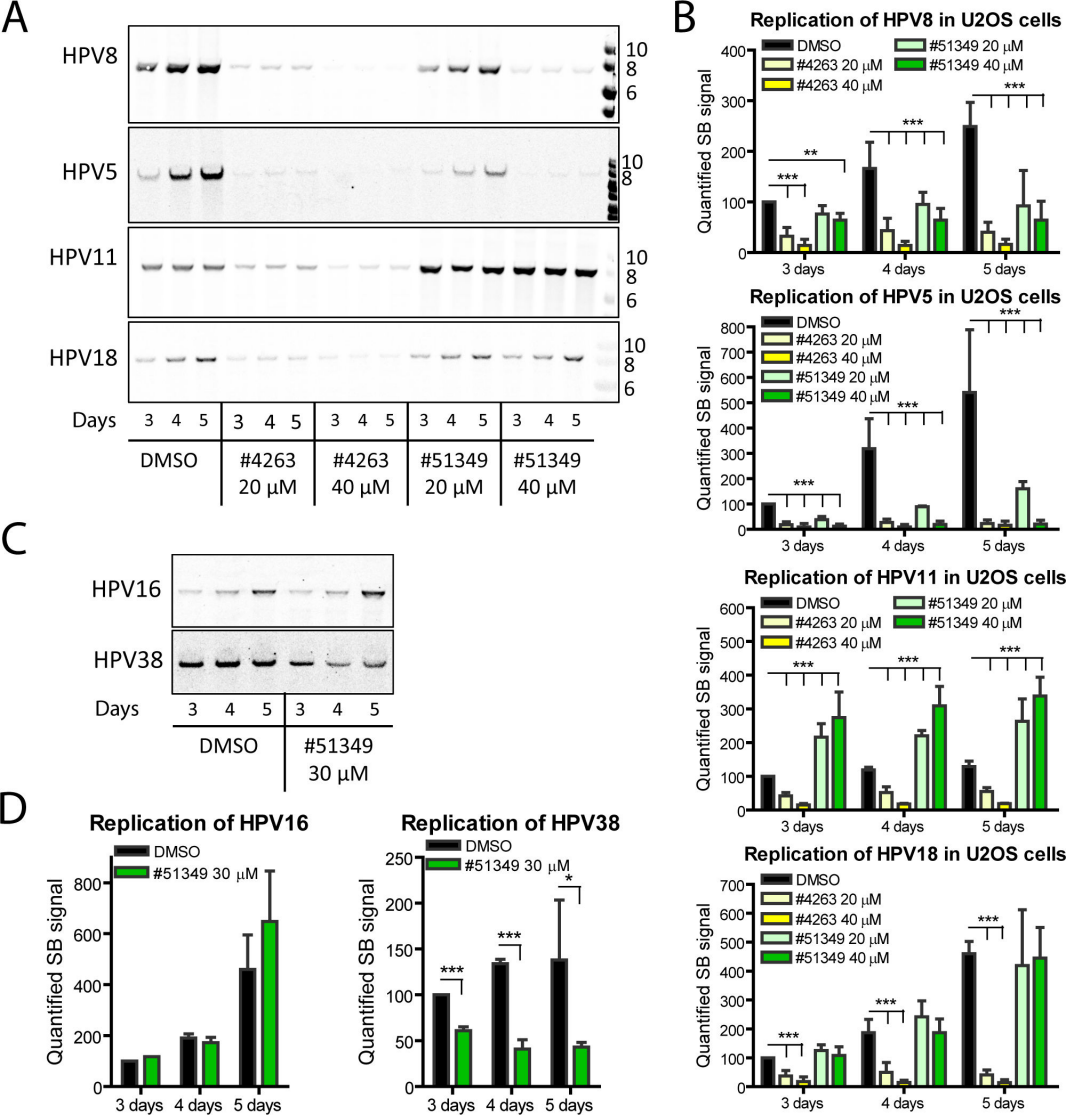
Transient replication of different HPV types in U2OS cells. (A–D) Replication of the cutaneous β-HPV types 5, 8, and 38, and mucosal α-types 11, 18, and 16 was analyzed using SB (**A, C**), and the signals corresponding to the replicated genomes were quantified (**B, D**). Chemicals were added to the cells one day after transfection, with DMSO used as a mock control. Total DNA was isolated at the indicated time points and treated with DpnI to digest the input viral DNA. HPV genomes were linearized using the appropriate restriction enzymes, transferred to a membrane, and hybridized with the corresponding radioactively labeled probes. The signals obtained on the third day post-transfection in the control samples treated with DMSO were set to 100%, and data from other samples were calculated relative to this control. All graphs: data are presented as the average percentage of at least three independent experiments ± SD; *P < 0.05, **P < 0.01, *** *P* < 0.001.

To analyze the inhibition spectrum of the selected chemicals, replication of cutaneous HPV types 5 and 8, as well as mucosal HPV types 11 and 18, was assessed in U2OS cells using Southern blotting (SB) (Fig. 2A). Both chemicals were used at concentrations of 20 and 40 µM, which substantially exceeded the IC_50_ values obtained in the Nluc assay to ensure visibility of the expected inhibitory effects and eliminate the need for daily medium replacement. Our analysis revealed that compound NSC4263 inhibited the replication of all tested HPV types, whereas compound NSC51349 specifically inhibited only cutaneous β-types 5 and 8. To confirm the selectivity of compound NSC51349 for cutaneous HPVs, we analyzed the replication of the highly oncogenic mucosal α-HPV type 16 and cutaneous β-HPV type 38 in the presence of 30 µM NSC51349, corroborating the results observed for other α- and β-HPV types (Fig. 2C).

To quantify the effects of the inhibitors, all SB panels from at least three independent experiments were analyzed. Replication signals were normalized to 100% in control samples treated with the diluent DMSO for 3 days ( Fig. 2B and 2D). Compound NSC4263 demonstrated similar inhibition across all tested HPV types, showing over 90% inhibition at 40 µM. The calculated IC_50_ values for HPV types 8, 5, 11, and 18 were 15 ± 1.2 µM, 14 ± 0.5 µM, 16 ± 0.3 µM, and 16 ± 1.3 µM, respectively, on the 3rd day of treatment (4th day post-transfection). Interestingly, compound NSC51349 upregulated the replication of HPV11 by approximately twofold and had no effect on the replication of HPV18 and HPV16. However, HPV5 replication was suppressed by approximately 70% and over 90% at 20 µM and 40 µM NSC51349, respectively. The effect of NSC51349 on HPV8 was slightly less pronounced, with replication signals decreasing by approximately 65% and 82% at 20 µM and 40 µM, respectively. Over time, the replication signals for HPV5 and HPV8 remained stably low in the presence of 40 µM NSC51349 (Fig. 2B, dark-green bars), and their increase was substantially inhibited by 20 µM NSC51349 (Fig. 2B, light-green bars). HPV38 replication was inhibited by approximately 65%, which may be due to its generally poor replication even in control cells treated with DMSO (Fig. 2D). Collectively, our data indicate that NSC51349 specifically inhibits the replication of cutaneous HPV types 5, 8, and 38 in U2OS cells.

### Compounds NSC4263 and NSC51349 exhibit different effects on U2OS proliferation

**Fig 3:**
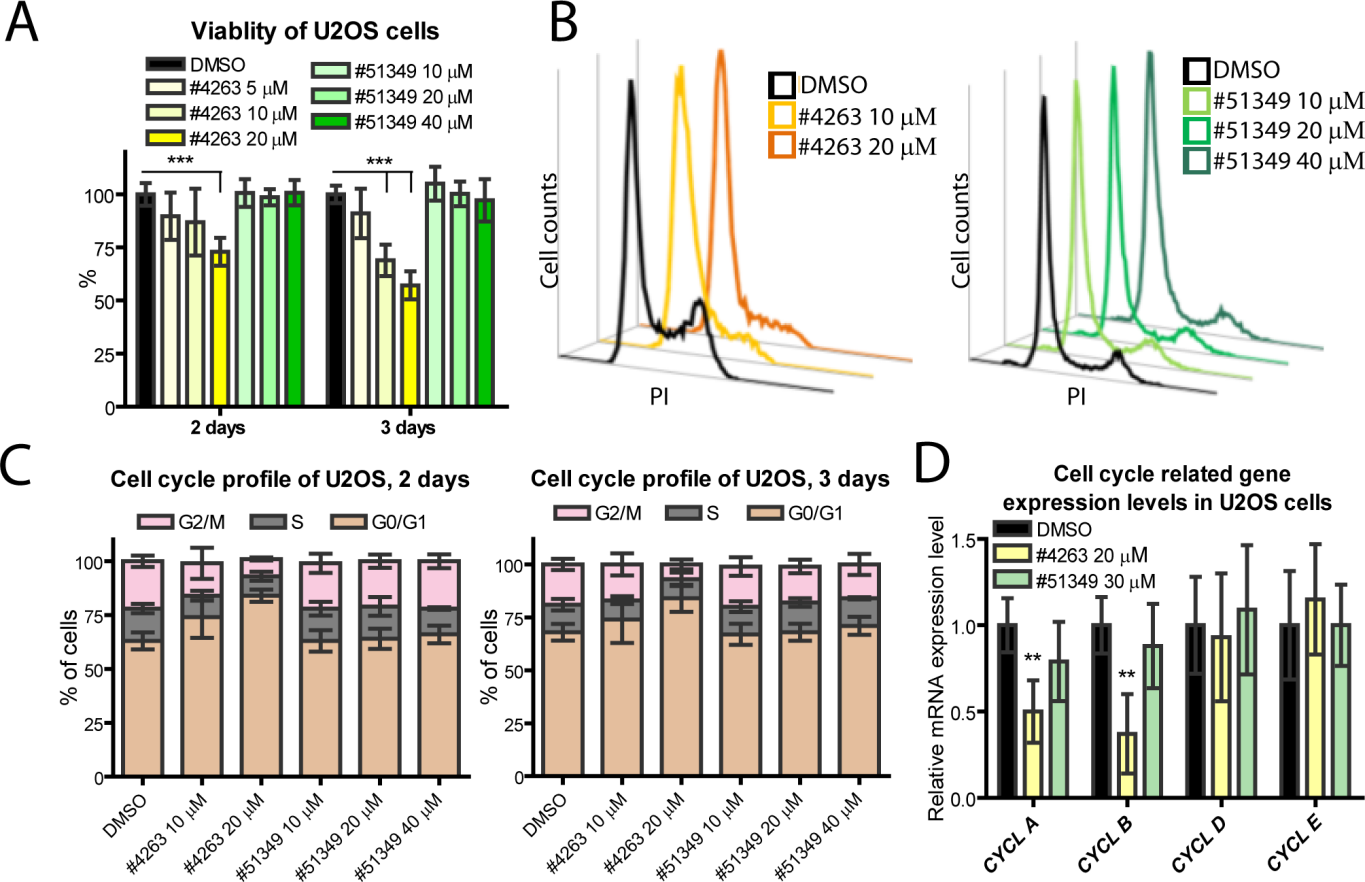
Viability and cell cycle profile of U2OS cells. (A) Viability of U2OS cells incubated with different concentrations of the inhibitors or DMSO for 2 or 3 days was assessed using an MTS assay. (B, C) U2OS cells were treated with different concentrations of the inhibitors and incubated for the indicated periods. The cell cycle profile was analyzed by flow cytometry using propidium iodide (PI) staining. (D) Expression levels of *cyclin (CYCL) A*, *B*, *D*, and *E* were measured using qRT-PCR, normalized to the expression levels of the housekeeping genes *GAPDH* and *ACTB*, and set to 1 in the control samples treated with DMSO. Data from other samples are expressed relative to this control. All panels: Data are presented as the mean of at least three independent experiments ± SD (**P < 0.01, ***P < 0.001).

To investigate potential cytotoxic effects of the identified inhibitors, we analyzed the viability of U2OS cells treated with different concentrations of NSC4263 and NSC51349 for 2 and 3 days using WST8- and MTS-based proliferation assays, which yielded similar results (Fig. 3A; data from the MTS assay are shown). The number of viable cells in the control sample treated with DMSO for 2 or 3 days was set to 100%. Our analysis revealed that NSC4263 inhibited the proliferation of U2OS cells in a concentration-dependent manner. Compared to the control, the number of viable cells decreased by 25% and 50% on the 2nd and 3rd days, respectively, with 20 µM NSC4263. In addition, a significant decrease in viable U2OS cells (approximately 30%) was observed with 10 µM NSC4263 on the 3rd day of treatment. By contrast, compound NSC51349 did not affect U2OS cell viability (Fig. 3A, green bars).

The observed decrease in viable cells treated with NSC4263 may result from either direct cytotoxicity or a deceleration of the cell cycle and reduced cell proliferation. To distinguish between these possibilities, we analyzed the U2OS cell cycle profile on the 2nd and 3rd days of treatment with different concentrations of NSC4263 or NSC51349 using propidium iodide and flow cytometry (Fig. 3B and C). Our analysis showed a robust shift toward the G0/G1 phases in cells treated with NSC4263. The number of cells in the G0/G1 phases increased approximately 11% and 20% in response to 10 and 20 µM NSC4263, respectively. By contrast, 10 and 20 µM NSC51349 had no significant effect on the U2OS cell cycle. However, a slight increase—approximately 3% and 7%—in the number of G0/G1-positive cells at the expense of G2/M cells was observed on the 2nd and 3rd days of treatment with 40 µM NSC51349, which prompted us to reduce the working concentration of the inhibitor to 30 µM in further short-term experiments (up to 5 days of the treatment) and to 20 µM in long-term experiments with U2OS cells or their derivates.

To strengthen these results, we analyzed mRNA expression levels of cell-cycle-related *cyclins A*, *B*, *D*, and *E* in U2OS cells treated with 20 µM NSC4263 or 30 µM NSC51349 for 2 days. Cyclin A regulates transition into the S and M phases in association with CDK2 and CDK1, respectively, with its transcription peak in the late S-/mid-G2 phase (21). Cyclin B is a mitotic cyclin required for progression through the M phase. Cyclins D and E are involved in the G1/S phase transition, with their synthesis beginning during the G1 phase. The levels of *cyclin A, B, D*, and *E* mRNAs, normalized to *GAPDH* and *ACTB*, are shown in Fig. 3D. Our qRT-PCR data indicated that *cyclins A* and *B*, which are associated with the S and G2/M phases, were significantly downregulated by approximately twofold in response to NSC4263 treatment, while mRNA levels of *cyclins D* and *E* remained similar to those in the DMSO-treated control cells. By contrast, compound NSC51349 did not alter the transcription of the analyzed cyclins. Collectively, our data demonstrate that compound NSC4263 inhibits the proliferation of U2OS cells by inducing G0/G1 arrest of the cell cycle, thereby characterizing its effects on HPV genome replication as indirect, occurring in a deregulated cell cycle-dependent manner. By contrast, compound NSC51349 has no adverse effect on the U2OS cell cycle, proliferation, or viability, suggesting that it directly interferes with the replication of β-HPV genomes.

### Compound NSC51349 specifically inhibits replication of HPV5-Nluc in primary keratinocytes

**Fig 4:**
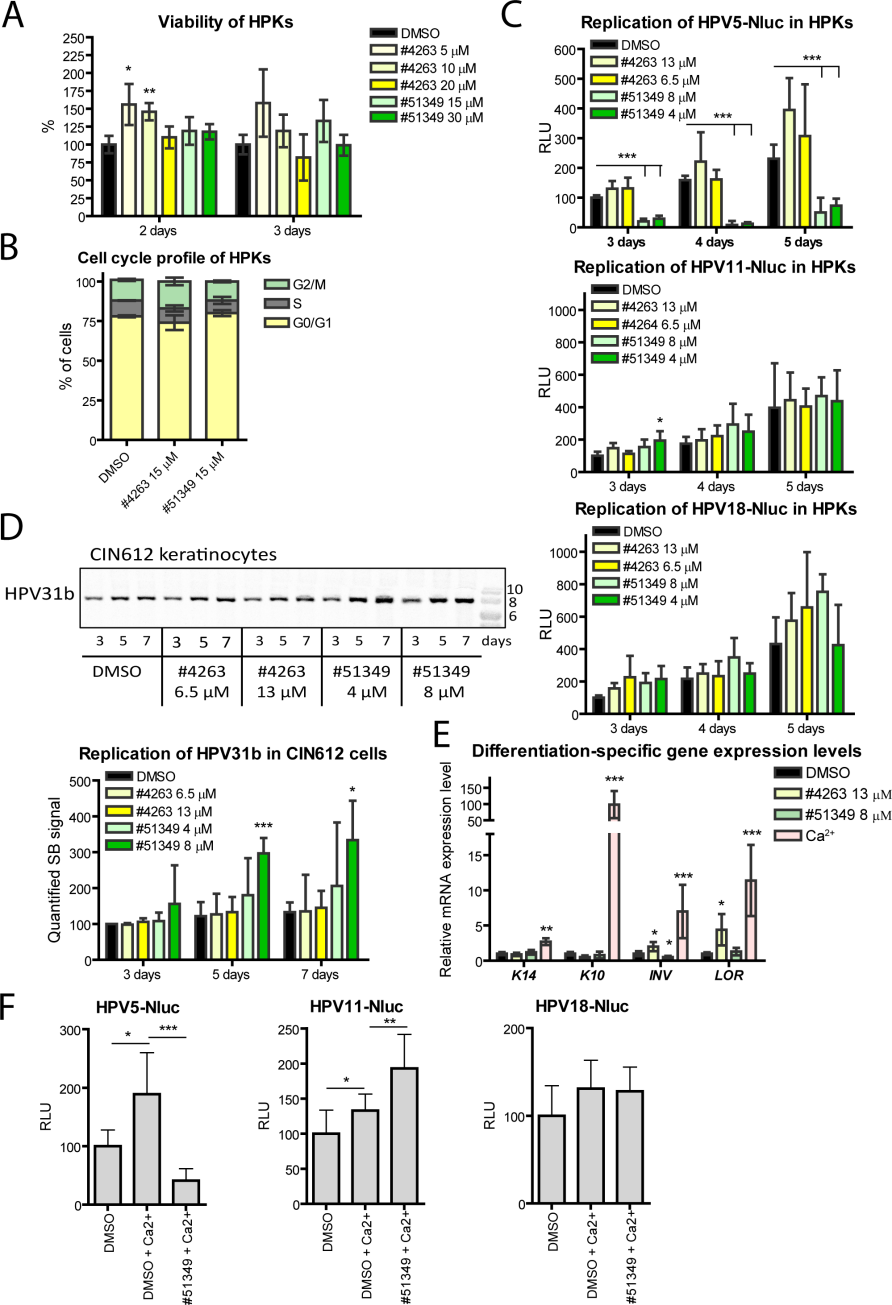
Replication of different HPV genomes in HPKs. (A, B) HPKs were treated with different concentrations of the compounds or DMSO for 3 days. The number of viable cells was measured using an MTS assay and set to 100% in the control sample treated with DMSO. The number of viable cells in other samples was calculated relative to this control. The cell cycle profile was analyzed by flow cytometry using propidium iodide staining. (C) HPKs were transfected with HPV genomes expressing Nluc and treated the next day with different concentrations of the compounds or DMSO in quadruplicate. Nluc activity was normalized to total protein concentrations. Normalized Nluc activity was set to 100% in cells treated with DMSO and incubated for 3 days, with data from other samples expressed relative to this control (RLU, relative luminescence units, days post-transfection are indicated). (D) CIN612E keratinocytes were treated with the indicated concentrations of the reagents for 3, 5, or 7 days. Total DNA was digested with the HindIII restriction enzyme to linearize the HPV31b genome and subjected to SB analysis. Right panel: SB signals from three independent experiments were quantified and set to 100% in the sample treated with DMSO for 3 days. Data from other samples are presented relative to this control. (E) HPKs were treated as indicated for 3 days. Expression levels of the keratinocyte differentiation markers *cytokeratin 10* (K10)*, cytokeratin 14* (K14)*, involucrin* (*INV*)*,* and *loricrin (LOR*) were measured using qRT-PCR and normalized to the mRNA expression levels of the housekeeping genes *GAPDH* and *ACTB*. The data were calculated relative to the normalized expression level of each specific gene in control cells treated with DMSO (set to 1). (F) HPKs were transfected with the respective HPV genome, incubated for 2 days, and then treated with either 8  µM NSC51349 or DMSO, in combination with 1.5  mM CaCl₂ to induce differentiation, for an additional 2 days. This was followed by the Nluc assay, as described in panel B. All graphs: Data are presented as the average percentage of at least three independent experiments ± SD (*P < 0.05, **P < 0.01, ***P < 0.001, *n* ≥ 3).

Epithelial keratinocytes are the natural host cells for HPV, capable of supporting the replication of various HPV types *in vitro* over a limited number of passages. This provides an opportunity to confirm the HPV-specific inhibitory effects of the identified compounds. Given the generally higher sensitivity of primary cells, we analyzed the viability of primary keratinocytes (HPKs) treated with different concentrations of NSC4263 and NSC51349 using an MTS-based assay (Fig. 4A). Surprisingly, neither of the compounds affected HPK viability as observed in U2OS cells (Fig. 3A). In fact, treatment with 5 and 10 µM NSC4263 resulted in a slight increase in the number of viable HPKs. However, on the 3rd day of treatment, a 20% decrease in viable cells was observed with 20 µM NSC4263, indicating the need to reduce the concentration of NSC4263 in further experiments. The cell cycle profile of HPKs treated with 15 µM NSC4263 or NSC51349 for 3 days remained similar to that of control DMSO-treated cells (Fig. 4B). Overall, our data suggest that compared to U2OS cells, HPKs are less sensitive to compound NSC4263, and both compounds, at concentrations up to 15 µM, appear to be harmless to HPKs.

To investigate the spectrum of inhibition by the identified compounds on HPV replication in HPKs, we used the Nluc assay to quantify Nluc-positive genome copy numbers. HPKs were transfected with HPV5-, HPV11-, and HPV18-Nluc genomes and treated with different concentrations of NSC4263 and NSC51349. Nluc activity was measured 3, 4, and 5 days post-transfection and normalized to either total protein content or alkaline phosphatase activity, which indicates the number of viable cells in the samples (Fig. 4C). None of the compounds inhibited replication of HPV11 and HPV18. In addition, NSC51349 caused a slight, mostly statistically insignificant increase in HPV11 and HPV18 copy numbers (Fig. 4C, green bars). By contrast, NSC51349 efficiently inhibited the replication of the HPV5 genome, with an IC_50_ of 4 ± 1.3 µM, approximately twofold lower than obtained in U2OS cells.

We also analyzed the effects of the compounds on the stable replication of the HR mucosal HPV type 31b genome in CIN612 9E immortalized keratinocytes. The cells were treated with various concentrations of the compounds for up to 1 week, and HPV31b genome levels and its physical state were analyzed using SB (Fig. 4D, upper panel, and Fig. S2A, respectively). Neither NSC4263 nor NSC51349 inhibited HPV31b replication. Quantification of the HPV31b replication signals from three independent experiments revealed approximately a twofold increase in HPV31b copy numbers in response to NSC51349 (Fig. 4D, bottom panel). A similar effect was observed in HPV11 and HPV18 transient replication assays in HPKs (Fig. 4C) and U2OS cells (Fig. 2B, panel for HPV11), confirming the inefficacy of NSC51349 towards mucosal α-HPVs.

The HPV life cycle is closely linked to the keratinocyte differentiation program. Keratinocyte differentiation, regulated *in vivo* by Ca^2+^ gradients, is associated with a robust increase in HPV genome copy numbers, especially in pre-terminally differentiated cells prior to new virion packaging (1, 22). To further examine the biological activities of the studied drug candidates, we analyzed the differentiation-specific gene expression profiles in HPKs treated with either NSC4263 or NSC51349 at concentrations of 13 and 8 µM, respectively, or with 1.5 mM Ca^2+^ as a positive control. Expression levels of genes specific to different stages of keratinocyte differentiation—such as *Keratin 14* (*K14*, a marker of early progenitor cells), *Keratin 10* and *involucrin* (*K10* and *INV*, markers of early differentiation), and *loricrin* (*LOR*, a marker of late differentiation)—were measured using qRT-PCR and normalized to *GAPDH* and *ACTB* expression levels (Fig. 4E). As expected, Ca^2+^ significantly increased the expression of all differentiation-specific genes analyzed. Surprisingly, *INV* and *LOR* mRNA expression levels increased 2-fold and 4.4-fold, respectively, in response to 13 µM NSC4263 (Fig. 4E, yellow bars). By contrast, NSC51349 had no significant effect on differentiation-specific gene expression levels.

Finally, we analyzed the efficiency of NSC51349 on amplification of the HPV5-, HPV11-, and HPV18-Nluc viral genomes following differentiation. HPKs were seeded in 96-well plates, transfected with HPV-Nluc genomes, incubated for 2 days, and then treated with 1.5  mM CaCl₂ along with either DMSO or 8  µM NSC51349 for an additional 2 days. As assessed by luciferase assay, the copy numbers of all viral genomes increased in response to CaCl₂-induced differentiation (Fig. 4F). NSC51349 inhibited the amplification of HPV5-Nluc in differentiated HPKs, but had no inhibitory effect on the replication of HPV11- and HPV18-Nluc genomes under the same conditions.

It has been reported that the NCI plated sets, such as Mechanistic set III and Diversity set V, contain a number of compounds unsuitable for drug development (23). The first group, identified using a REOS (Rapid Elimination of Swill) filter, includes chemicals with undesirable physicochemical properties (e.g., lipophilicity, molecular weight, or hydrogen-bonding characteristics). Another group comprises Pan-Assay Interference Compounds (PAINs), which either react with multiple target proteins or disrupt the normal functioning or accuracy of the assay. Compound NSC51349 or 5,7-dimethoxy-2-pyridin-3-ylchromen-4-one passed both filters, making it an attractive candidate for further investigation. By contrast, compound NSC4263 or 5-Nitro-1,10-phenanthroline did not pass the REOS filter. This fact and the ability of compound NSC4263 to induce differentiation-specific *INV* and *LOR* gene expression in HPKs make it unsuitable for further drug development. Therefore, compound NSC4263 was excluded from further investigations.

### NSC51349 inhibits E1- and/or E2-dependent stable replication of HPV5

Stable maintenance of extrachromosomal HPV genomes is crucial for cellular transformation and cancer (1). It is attributable to long-term HPV infections that occur before and/or concurrently with HPV genome integration into the host cell chromosomes. Our data showed that compound NSC51349 upregulated episomal HPV31b genome copy numbers in CIN612 9E keratinocytes (Fig. 4D; Fig. S2A). Since there are no β-HPV-positive keratinocytes in our laboratory, we used U2OS-based stable cell lines bearing the HPV5 episomal genome to study the efficacy of the identified inhibitor in experimental settings of HPV stable replication (20, 24).

These HPV-positive U2OS-derived cells allow us to distinguish between E1-/E2-specific and host cell replication machinery-specific viral DNA replication by analyzing the different oligomeric forms of uncut HPV genomes (24, 25). We have demonstrated that the prevalent replicons of the episomal HPV5 and HPV18 genomes, comprising up to 80% of all viral DNA, replicate in an E1- and/or E2-independent manner in U2OS cell-derived stable cell lines (24–26). By contrast, replication of other forms of HPV DNA, including the ccc and oc forms, depends on E1 and/or E2 biological activities. These results were corroborated using the *E1* and *E2* RNAi and the previously described U2OS cells-derived cells stably bearing episomal HPV5 genome (20, 24, 26).

**Fig 5:**
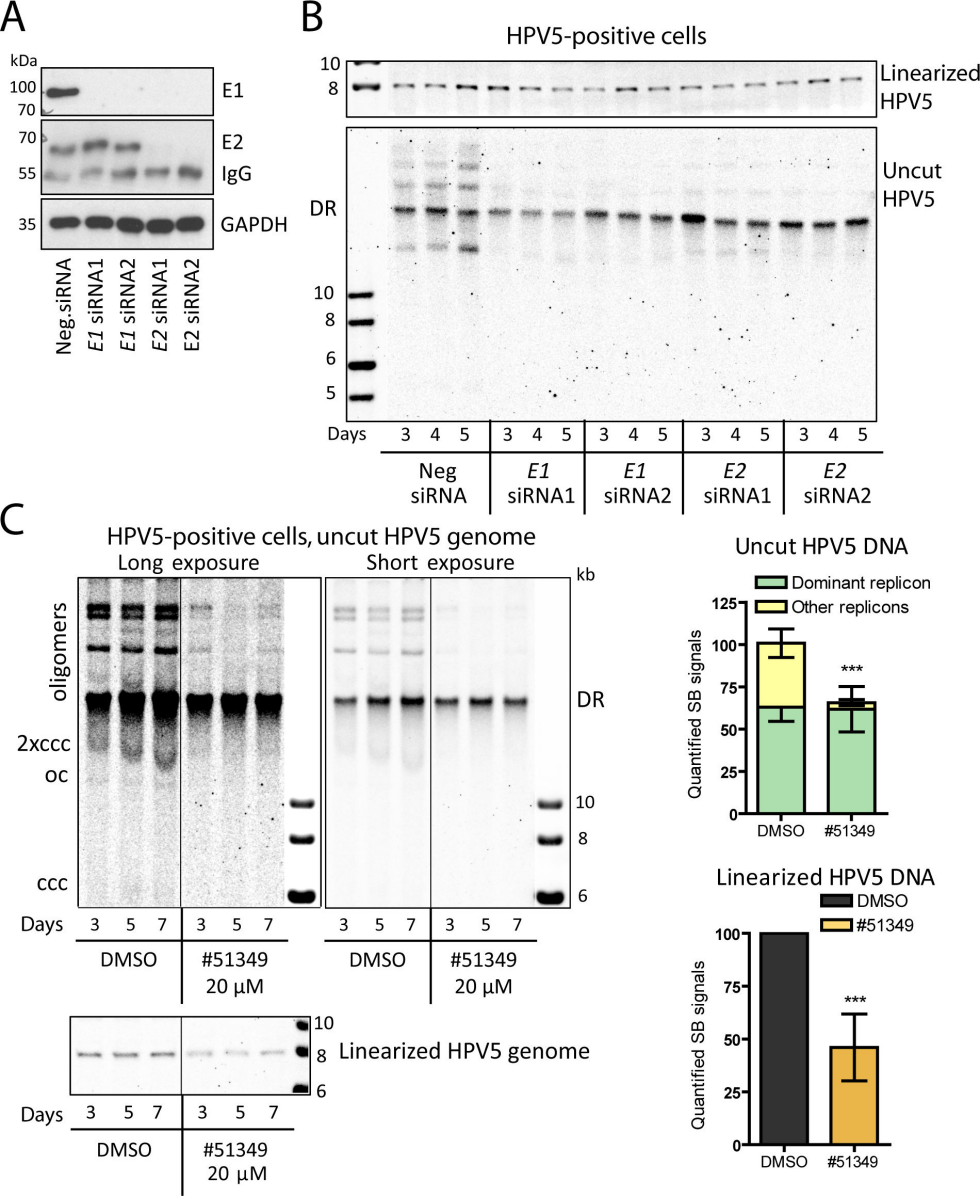
Replication of the HPV5 genome in a stable cell line is affected by compound NSC51349. (A) HPV5-E1HA-Nluc-E2Flag genome-positive U2OS cells were transfected with the indicated siRNAs. The E1 and E2 proteins were immunoprecipitated and analyzed using the HA- and E2-specific antibodies by immunoblotting. GAPDH was used as a loading control. (B) U2OS-derived cells bearing the HPV5 genome were treated with the indicated siRNAs and incubated up to 5 days. LMW DNA was isolated and treated with either the SacI restriction endonuclease to linearize the HPV5 genome or the HPV5-noncutting restriction enzyme NdeI, followed by SB analysis. (C) HPV5-positive cells were treated with 20 µM compound NSC51349 for 3, 5, and 7 days and subsequently analyzed as described in (B). Images taken after short and long exposure times are shown to distinguish the intensities of the dominant replicon (DR) signals and to visualize the covalently closed circular (ccc) and open circular (oc) forms of the viral genome, respectively. The signals corresponding to the linearized HPV5 genome were quantified and set to 100% for control cells treated with DMSO. Data from NSC51349-treated samples are presented relative to this control. For analysis of the uncut HPV5 genome pattern, SB signals corresponding to the oligomeric dominant replicon (DR) and other replicons were quantified. The sum of the pixels obtained from each sample was set to 100%, and the intensities of the HPV5 dominant form and all other replicons combined were calculated relative to the 100% in the control sample treated with DMSO. The intensities of the corresponding replicons in the NSC51349-treated samples are shown relative to the control (*** *P*  <  0.001, *n* = 3).

Two different *E1*- and *E2*-specific siRNAs were transfected into HPV5-E1HA-Nluc-E2Flag-positive U2OS cells, and E1 and E2 protein levels were analyzed by immunoblotting (Fig. 5A). All siRNAs effectively knocked down their specific targets. The absence of E1 protein expression in the case of *E2* siRNA transfection is due to E2 being a positive transcriptional regulator of *E1* expression. As expected, the *E1-* and *E2*-specific siRNAs efficiently inhibited *E1* and *E2* gene expression as well as copy numbers of the HPV5-E1HA-Nluc-E2Flag and HPV5 genomes in the transient replication assay in U2OS cells (Fig. S1A through C).

Viral genome copy numbers in HPV5-positive U2OS cells transfected with *E1*- or *E2*-specific siRNAs were assessed by analyzing LMW DNA via SB using either linearizing or non-cutting HPV5 DNA restriction enzymes (Fig. 5B). Analysis of the physical state of the HPV5 genome during both stable and transient replication revealed a pattern of mono- and oligomers of various sizes, similar to what has been previously described (24). In cells that stably maintain the HPV5 genome, we observed both monomeric and oligomeric forms of HPV5 DNA, including the previously described dominant oligomeric form—referred to as the dominant replicon (DR)—which was negligible during transient replication or in the input HPV5 DNA (Fig. S1D). In cells stably maintaining the HPV5 genome, *E1/E2* RNAi caused the disappearance of ccc and other minor forms of the viral genome (Fig. 5B). However, replication of the HPV5 DR remained largely unaffected by treatment with E1/E2 siRNAs, as determined by quantification of the corresponding SB signals (Fig. S1E, upper panel). These data indicate an E1-/E2-independent replication mechanism in cells stably maintaining the HPV5 genome. This behavior is similar to the previously reported stable replication of mucosal HPV18 (25). Quantification of the linearized HPV5 genome SB signals revealed an average decrease in viral genome copy numbers of approximately 23%, 40%, and 53% on the 3rd, 4th, and 5th days post-transfection with E1/E2 siRNAs, respectively, compared to cells treated with negative control siRNA at the corresponding time points (Fig. S1E, bottom panel).

Next, to analyze the effect of the NSC51349 compound on stable HPV5 replication, HPV5-positive cells were treated with 20 µM NSC51349 for up to 1 week, followed by SB analysis of LMW DNA treated with linearizing or non-cutting HPV5 DNA restriction enzymes (Fig. 5C). Quantification of the linearized HPV5 genome showed a twofold decrease in HPV5 copy numbers in response to NSC51349 treatment. Treatment with NSC51349 resulted in the inhibition of all HPV5 replicons except the dominant oligomeric form, as observed in the *E1/E2* RNAi experiment (Fig. 5B and C). The SB signals corresponding to different HPV5 replicons were quantified, and their sum was set as 100% in the control samples treated with DMSO. The DR accounted for approximately 60% of all viral DNA, and its intensity did not change significantly over the course of the experiment (Fig. 5C, right panel, green bars). By contrast, the intensity of all other forms of viral DNA decreased by more than 90% (Fig. 5C, right panel, yellow bars). Taken together, our results indicate that the inhibition of HPV5 replication induced by NSC51349 involves an E1- and/or E2-dependent mechanism.

### The mechanism of the NSC51349-mediated inhibition of HPV5 replication includes suppression of the E2 transcriptional activity

Analysis of the stably replicating HPV5 genome oligomers indicated that NSC51349 affects E1 and/or E2 biological activities. E1 is a helicase essential for HPV DNA replication, whereas E2 is a multifunctional protein involved in regulating transcription, replication, and chromosomal tethering of the viral genome (27, 28).

**Fig 6:**
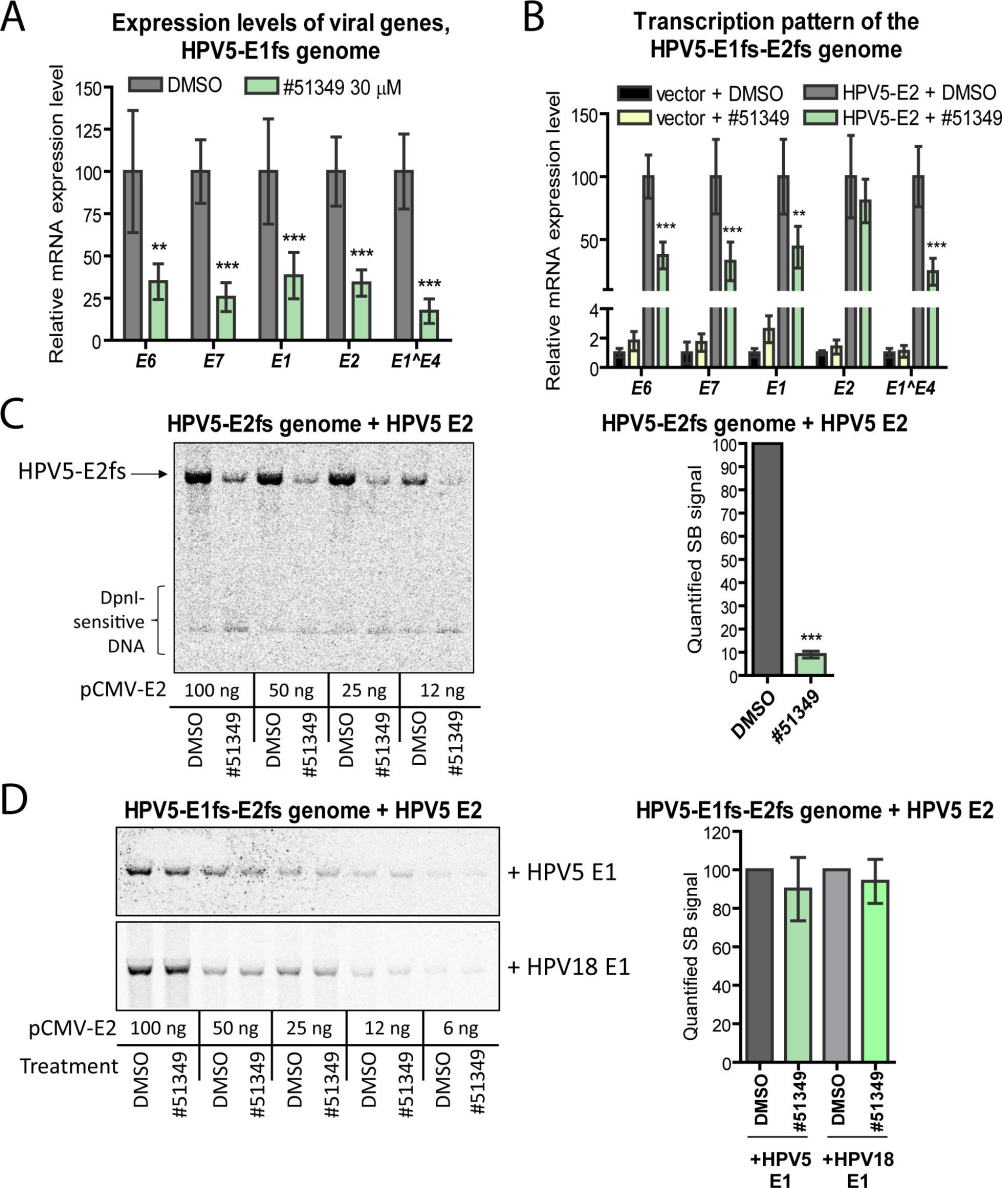
Mechanism of NSC51349-dependent inhibition of HPV5 replication. (A, B) U2OS cells were transfected with replication-deficient HPV5 genomes expressing either a C-terminally truncated E1 protein (HPV5-E1fs, A) or deficient in both E1 and E2 proteins (HPV5-E1fs-E2fs, B). To induce viral gene expression, the HPV5-E1fs-E2fs genome was co-transfected with either an HPV5 E2 expression construct or an empty vector as a control. The following day, cells were treated with either DMSO or 30 µM NSC51349 for 24 h. The levels of respective viral gene mRNA expression were measured by qRT-PCR in triplicate using two different pairs of primers, normalized to *GAPDH* mRNA expression levels, and set to 1 in the control samples treated with DMSO. Data from other samples are expressed relative to the control. (C) U2OS cells were transfected with the replication-deficient HPV5-E2fs genome expressing WT E1. To induce viral genome transcription and replication, different amounts of the HPV5 E2 encoding plasmid were co-transfected. Cells were treated with either 30 µM NSC51349 or DMSO for 24 h. The level of the replicated HPV5-E2fs genome was analyzed using SB (left panel), quantified, and set to 100% in the control samples treated with DMSO (right panel). The image was processed in Photoshop to enhance the visualization of bands corresponding to DpnI-sensitive input DNA. (D) U2OS cells were transfected with the HPV5-E1fs-E2fs genome expressing truncated forms of biologically inactive E1 and E2 proteins, resulting in a deficiency in viral transcription and replication. To induce replication of the viral genome, plasmids encoding HPV5 E1 and different amounts of HPV5 E2 were co-transfected. Alternatively, a plasmid encoding HPV18 E1 was co-transfected instead of HPV5 E1 to examine the role of the E1 protein in the NSC51349-mediated inhibition of HPV5 genome replication. The following day, cells were treated with DMSO or NSC51349 for 24 h. Total DNA was isolated, treated with DpnI to digest input DNA and SacI to linearize the HPV5 genome, and analyzed using SB (left panel). The SB signals were quantified and expressed as the average percentage of at least three independent experiments (right panel). All graphs: data are presented as mean ± SD, **P < 0.01, *** *P* < 0.001.

To determine whether NSC51349 affects E2 transcriptional activity, we analyzed the expression levels of viral early transcripts in U2OS cells treated with NSC51349. To prevent contamination of complementary DNA with viral DNA replicons and to avoid transcription from the replicated copies of the transfected genome, we used the replication-deficient HPV5-E1fs genome, which expresses WT E2 but has a truncated, inactive E1 due to a frameshift in its ORF (29). U2OS cells were transfected with the HPV5-E1fs genome, and the following day, 30 µM NSC51349 was added for 24 h. Our qRT-PCR analysis showed that treatment with NSC51349 resulted in a significant decrease (approximately 70%) in the expression levels of all viral genes analyzed (Fig. 6A).

HPV transcription, primarily controlled by the E2 protein, is also regulated by cellular transcription factors that bind to specific sequences within the viral upstream regulatory region (URR) (30). The observed decrease in viral gene expression levels may be due to altered E2 activity and/or the activity of other cellular proteins involved in HPV transcriptional regulation. To distinguish between these possibilities, we used the HPV5-E1fs-E2fs genome (31). This genome, due to the knockout of both E1 and E2, is deficient in both replication and E2-dependent transcription but remains permissive for E2-independent transcriptional regulation by host cell proteins. U2OS cells were co-transfected with the HPV5-E1fs-E2fs genome and either an empty vector or an HPV5 E2 expression plasmid. The following day, the cells were treated with NSC51349 or DMSO for 24 h, and levels of viral transcripts were measured using qRT-PCR. The normalized mRNA expression level of each viral gene triggered by the overexpressed E2 in the cells treated with DMSO was set as 100%, and data from other samples were expressed relative to this control.

Our analysis showed that although the overall level of endogenous E2-independent transcription was low, it was detectable but significantly unaffected by NSC51349 (Fig. 6B, yellow versus black bars). Overexpression of E2 resulted in a robust increase in the expression levels of all early viral genes, which was significantly suppressed (approximately 55%) by NSC51349 (Fig. 6B, gray versus green bars). These data indicate that NSC51349 inhibits the transcriptional activity of overexpressed E2 protein, although to a lesser extent compared to its effect on endogenous E2 (Fig. 6A). This difference may be attributed to the different amounts of overexpressed and endogenous proteins, as well as the transcriptional self-regulation of endogenous E2.

Next, we analyzed whether NSC51349 inhibits only E2 transcriptional activity or if it also affects other functions of E2 or the activity of E1. To distinguish between these possibilities, we used two modified HPV5 genomes: HPV5-E2fs, which is deficient in endogenous E2 but expresses WT E1, and the previously described HPV5-E1fs-E2fs. In the case of HPV5-E2fs, the overexpressed E2 protein acts as a regulator of both transcription and replication; it induces the expression of endogenous E1 and participates in viral genome replication. For HPV5-E1fs-E2fs, the transcriptional activity of overexpressed E2 is dispensable due to a frameshift in the *E1* ORF, and E1 must be added *in trans* to trigger viral genome replication. In this case, E2 acts solely as a replication factor (29, 31).

We co-transfected either HPV5-E2fs or HPV5-E1fs-E2fs genomes with different amounts of the E2-encoding plasmid into U2OS cells. For HPV5-E1fs-E2fs, a constant amount of an HPV5 E1-encoding construct was included in all samples. Two days post-transfection, we analyzed replication efficiencies of the HPV5-E2fs and HPV5-E1fs-E2fs genomes in cells treated with NSC51349 or DMSO for 24 h (Fig. 6C and D, respectively). Our analysis showed that the overexpressed E2 was able to trigger transcription and replication of the HPV5-E2fs genome in a concentration-dependent manner (Fig. 6C, left panel). A similar sequential increase in replication signals was observed for HPV5-E1fs-E2fs (Fig. 6D, left upper panel). However, treatment with NSC51349 yielded dramatically different results: replication of the HPV5-E2fs genome was almost completely suppressed, whereas replication of HPV5-E1fs-E2fs was not notably affected. Quantification of the replication signals from three independent experiments revealed approximately 90% inhibition for HPV5-E2fs and up to 10% inhibition for HPV5-E1fs-E2fs (Fig. 6C and D, right panels). These results indicate that NSC51349 has no significant effect on E2 biological activities required for viral genome replication but efficiently inhibits E2 transcriptional activity.

It has been shown that β-HPV-derived E2, in combination with both β- and α-HPV-derived E1 proteins, can trigger replication of plasmids containing viral URRs (32). Our results demonstrated that NSC51349 had no effect on the replication of HPV18, indicating that the HPV18 E1 and E2 proteins are insensitive to this compound (Fig. 2A and B). To test whether HPV5 E1 activity is affected by NSC51349, we analyzed the replication of the HPV5-E1fs-E2fs genome co-transfected with increasing amounts of HPV5 E2 and a constant amount of HPV18 E1. We challenged the transfected cells with NSC51349 for 24 h and compared the replication signals to those obtained with HPV5 E1 in the same experimental settings (Fig. 6D, left lower and upper panels, respectively). Similar results were observed with both HPV18 E1 and HPV5 E1. Quantification of the SB signals revealed that, compared to the respective DMSO-treated controls, HPV5 E1-driven replication of the HPV5-E1fs-E2fs genome was 90% ± 16%, while replication in the presence of HPV18 E1 was 94% ± 11% in cells challenged with NSC51349 (Fig. 6D, right panel). Taken together, these observations confirm that NSC51349 specifically targets the E2 transcription factor while leaving HPV5 E1 biological activity intact.

### NSC51349 inhibits replication of the *Macaca fascicularis* PVs

**Fig 7:**
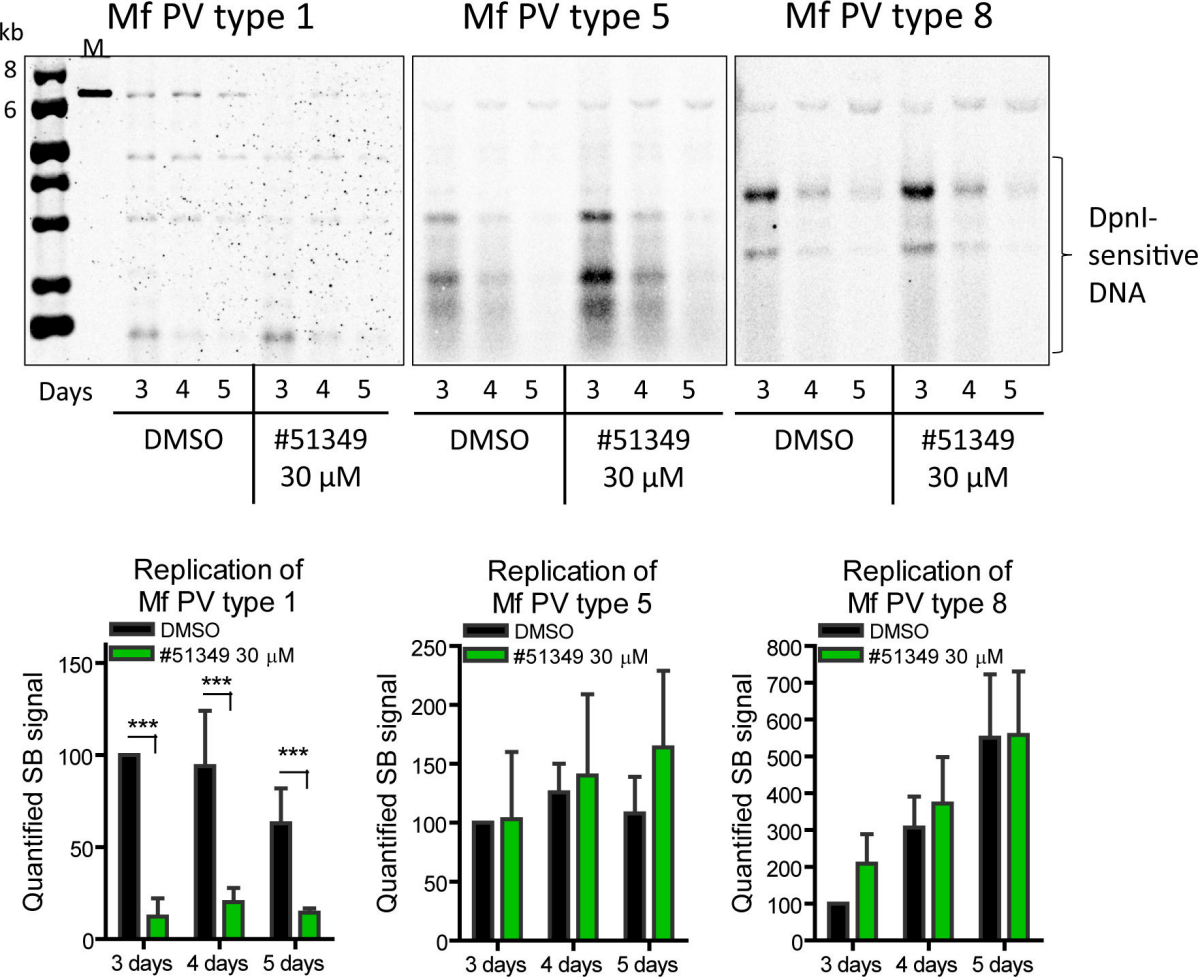
Replication of the M. *fascicularis* PV types 1, 5, and 8 genomes in U2OS cells was transfected with the indicated genomes and treated with either NSC51349 or DMSO. LMW DNA was isolated, treated with DpnI and restriction enzymes to linearize the PV genomes, and analyzed using SB (with days post-transfection indicated). LMW bands correspond to DpnI-sensitive input DNA (upper panels). Bottom panels: the signals corresponding to the replicated genomes were quantified and set to 100% for samples treated with DMSO for 2 days (3 days post-transfection). The data from other samples were calculated relative to this 100%. Data are presented as average mean ± SD, ***P < 0.001, *n* = 3.

Testing drug candidates using *in vivo* models is the final preclinical phase of drug development. *M. fascicularis* PVs type 1, 5, and 8 genomes have previously been described as valuable experimental tools that enable the use of *in vivo* models for cervical, cervicovaginal, and cutaneous infections in antiviral drug development (33). The Mf PV1, isolated from skin, is similar to cutaneous β-HPVs, while Mf PV types 5 and 8 are closer to mucosal α-HPVs. It has been shown that Mf PVs and their mutants deficient in the E8 repressor replicate in U2OS cells, and we utilized these genomes to test the NSC51349 drug. U2OS cells were transfected with Mf PV genomes and treated with NSC51349 for 3, 4, and 5 days. Replication of the viral genomes was analyzed using SB (Fig. 7). The NSC51349 compound inhibited the replication of cutaneous Mf PV1 genome more than 90%, while replication of Mf PV5 and Mf PV8 was not significantly affected. These data suggest that *M. fascicularis* and Mf PV1 have potential for further use in *in vivo* models of cutaneous infections. The physical state of the *M. fascicularis* PV genomes during transient replication in U2OS cells was analyzed using SB and LMW DNA isolated on the 3rd and 5th day post-transfection (Fig. S2B). Our results showed that transient replication of all forms of the Mf PV1 genome was inhibited by NSC51349 in a similar manner, while its effect on replication of the mono- and oligomeric forms of Mf PV types 5 and 8 was negligible.

### Molecular docking predicts E2-NSC51349 interaction

**Fig 8:**
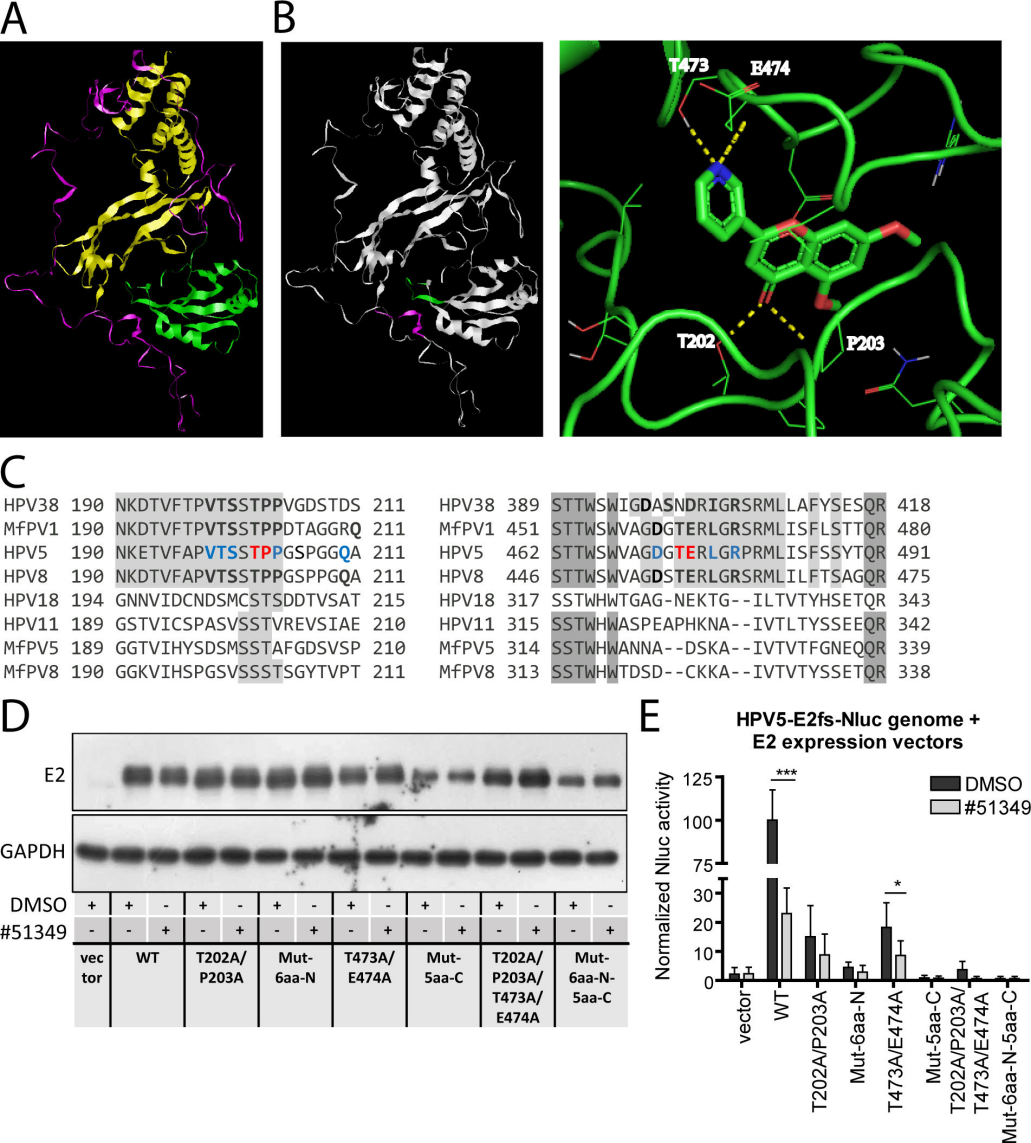
Computational analysis of the HPV5 E2 protein structure and E2-NSC51349 interaction. (A) The structure of the HPV5 E2 protein was predicted using AlphaFold with MMseqs2 MSA and minimized using a 30 ns molecular dynamics (MD) simulation. The N-terminal TAD, corresponding to amino acid residues 1–200, is highlighted in yellow, the C-terminal DNA-binding domain is shown in green, and the hinge region is displayed in magenta. (B) AutoLigand was used to predict the NSC51349-binding site in the E2 protein. The binding was predicted to occur between amino acids 198–210 and 471–478, which are highlighted in magenta and green, respectively (left panel). The binding pose at the predicted binding site of E2 was calculated by the AutoDock Vina 1.1.2 engine. Possible hydrogen bonds are shown as yellow dashed lines (right panel). (C) An alignment of the sequences of the E2 proteins derived from NSC51349-sensitive and -insensitive PV types analyzed in the present study was performed using the Clustal Omega algorithm. Conserved residues are highlighted in gray. The residues T202, P203, T473, and E474, predicted to be directly involved in NSC51349 ligand binding, are shown in red, and other residues potentially involved in the interaction are shown in blue. The corresponding residues in other NSC51349-sensitive PV types are shown in bold. (D, E) U2OS cells were transfected with the HPV5-E2fs-Nluc genome and expression constructs encoding the indicated HPV5 Flag-tagged E2 proteins or an empty vector. The next day, cells were treated with DMSO or 20 µM NSC51349 for 24 h. Expression levels of the E2 proteins were analyzed by immunoblotting using a Flag-HRP antibody. GAPDH was used as a loading control (**D**). (**E**) Nluc activity was normalized to alkaline phosphatase activity, and the normalized Nluc activity in control cells transfected with the WT E2 and treated with DMSO was set to 100%. All other data were calculated relative to the control sample. Data are presented as the average percentage of at least three independent experiments ± SD; *P < 0.05, ***P < 0.001.

To further characterize the interaction between the identified inhibitor NSC51349 and the HPV5 E2 protein, and to map a potential binding site, we first generated a computationally predicted structure of the E2 protein using AlphaFold with MMseqs2 MSA. This structure was subsequently minimized using molecular dynamics (MD) simulation by the Desmond program package of Schrödinger LLC and used for further molecular docking (34). The Rasmol viewer was employed to visualize the E2 protein structure and its three distinct domains: the N-terminal transactivation domain (TAD, comprising the first 200 amino acids), the C-terminal DNA-binding/dimerization domain (approximately 100 amino acids), and the unstructured hinge region connecting these domains (Fig. 8A). The Ramachandran plot indicates that most of the structurally resolved protein is present in the most favored conformations (Fig. S2C, black dots in the red regions). This implies that the protein’s backbone dihedral angles predominantly fall within the sterically favorable regions, which correspond to common secondary structures like α-helices and β-sheets.

Potential binding sites were identified using AutoLigand software (35). Next, the AutoDock Vina 1.1.2 molecular docking engine was used to predict the potential binding mode of NSC51349 to E2. The molecular docking results were analyzed using PyMOL software. The most favorable docking pose, with a binding energy of −7.4 kcal/mol, is shown in Fig. 8B (right panel). The amino acid residues predicted to interact with NSC51349 via hydrogen bonds include threonine 202 (T202), proline 203 (P203), threonine 473 (T473), and glutamate 474 (E474). In addition, several nearby residues—such as valine 198 (V198), threonine 199 (T199), serine 200 (S200), proline 204 (P204), asparagine 471 (D471), leucine 476 (L476), and arginine 478 (R478)—were predicted to contribute to the interaction with the ligand. These residues are located at the junction of the E2 TAD and hinge region, as well as in the C-terminal part of the DNA-binding domain, which are situated in close proximity to each other according to the simulated E2 3D structure (Fig. 8B, right panel). To visualize their spatial arrangement, peptides 198–210 and 471–478 are highlighted.

A Clustal Omega sequence alignment of E2 proteins from the HPV types analyzed in this study revealed that the identified residues are part of highly conserved motifs in NSC51349-sensitive PV types, such as HPV5, HPV8, Mf PV1, and HPV38 (Fig. 8C). Moreover, our bioinformatic analysis showed that the V/ITSST/SP motif is conserved in 92% of β-HPV E2 proteins (49 out of 53), with the remaining four E2 proteins displaying only a single amino acid substitution. Proline residue 204 is conserved in 75% of β-HPV E2. Similarly, the D/ERV/I/LGR motif is conserved in 92% of β-HPV E2 proteins, with only four E2 proteins containing A or T instead of D/E. The least conserved residue corresponds to T473 in HPV5 E2, with either T or S present in 32% of the sequences. In contrast to β-HPVs, these motifs are either less conserved or absent in NSC51349-insensitive HPV types 18 and 11, as well as Mf PV types 5 and 8 (Fig. 8C). Taken together, these data provide deeper insights into the E2-NSC51349 complex formation, which may help to explain the mechanism of NSC51349-mediated inhibition and to predict its inhibitory spectrum.

To confirm our molecular docking results, we mutated the key amino acid residues predicted to interact with the ligand to alanine (Table 1). Constructs expressing Flag-tagged WT and mutant E2 proteins (or the respective empty vector) were co-transfected with the replication-deficient HPV5-E2fs-Nluc genome into U2OS cells. The next day, the cells were treated with DMSO or NSC51349 for 24 h. E2 protein levels were assessed using immunoblotting with an anti-Flag-HRP antibody, and the efficiency of viral genome replication was evaluated using an Nluc assay (Fig. 8C and D).

**TABLE 1:** HPV5 E2 mutant proteins

Construct	Mutated amino acid residues
E2-T202A/T203A	T202, T203
E2-Mut-6aa-N	V198, T199, S200, T202, P203, P204
E2-T473A/E474A	T473, E474
E2-Mut-5aa-C	D471, T473, E474, L476, R478
E2-T202A/T203A/T473A/E474A	T202, P203, T473, E474
E2-Mut-6aa-N-5aa-C	V198, T199, S200, T202, P203, P204, D471, T473, E474, L476, R478

Quantification of the WB results showed that the levels of all overexpressed E2 proteins were similar across samples, except for E2 proteins bearing five substitutions in the C-terminal domain (D471, T473, E474, L476, and R478) (Fig. S2D). The expression level of these proteins was approximately 40% lower compared to the WT E2. The inhibitor NSC51349 had no negative effect on E2 protein expression levels.

However, all mutant E2 proteins exhibited significantly lower biological activity compared to the WT E2 (Fig. 8D). Proteins with two mutations, either T202/P203 or T473/E474, demonstrated approximately 20% residual activity, indicating that their ability to support HPV5-E2fs-Nluc genome replication was reduced by more than 80%. The activity of all other E2 mutants was negligible, with Nluc activity in these samples similar to that of the empty vector.

The inhibitor NSC51349 reduced the WT E2 biological activity approximately fivefold or approximately 80%, while inhibition of the E2-T202/P203 and E2-T473/E474 mutant proteins was less efficient—43% and 53%, respectively. No inhibition was observed for biologically inactive E2 mutant proteins. These data indicate that the amino acids conserved in the β-HPV E2 proteins, which are predicted to interact with the inhibitor, are essential for E2 biological activity. In addition, the observed reduction of at least twofold in NSC51349 inhibitor efficiency toward the mutant proteins proves that these amino acids play a role in ligand interaction.

## DISCUSSION

HPV infections can lead to a wide range of epithelium-associated pathologies. While prophylactic vaccines have been developed to combat HPV infections, they target only a small subset of mucosal HPV types and are ineffective against ongoing infections. Therefore, there is a clear unmet medical need for the development of specific HPV antiviral drugs.

One potential viral process to target with drugs is the replication of viral genomes. The rationale behind this approach is that reducing the viral load and decreasing the expression of viral oncogenes E6 and E7 is particularly important in HPV infections, where viral genomes replicate as multicopy episomes in the nuclei of infected cells during persistent infection. However, targeting viral genome replication may not be the most effective strategy for mucosal HPV infections, which can lead to cervical and head-and-neck cancers, as the viral genome often integrates into the host cell chromosomes. By contrast, cutaneous HPV types that infect the epithelium generally do not integrate but can still cause various medical conditions requiring treatment.

In the present paper, two compounds capable of inhibiting the replication of the cutaneous β-HPV5 genome were identified through HTS of the NCI Diversity Set VI library of small-molecule compounds in U2OS cells. One of these compounds, 5-Nitro-1,10-phenanthroline (NSC4263), was effective against all HPV types tested (cutaneous HPV types 5 and 8, mucosal HPV types 11 and 18), thus representing a pan-HPV replication inhibitor. However, subsequent analysis revealed that while it inhibited HPV replication, it also caused dramatic G0/G1-phase cell cycle arrest in U2OS cells and negatively affected the viability of these cells. Interestingly, in contrast to U2OS cells, the NSC4263 compound had no effect on the cell cycle or viability of HPKs, nor did it inhibit the replication of any of the tested HPV genomes (HPV types 5, 11, and 18) in these cells. Similarly, the NSC4263 compound was ineffective in inhibiting the replication of mucosal HPV type 31b in CIN612 keratinocytes.

5-Nitro-1,10-phenanthroline is a derivative of 1,10-phenanthroline (phen), with an additional nitro group substitution in the central aromatic ring (Fig. 1C). Phen is a well-known chelator that can inhibit various metal-dependent enzymes by chelating the metal ion away from the enzyme’s active center. In addition, it has been shown that phen may act as an intercalating molecule for nucleic acids and promote DNA damage, which can lead to cell cycle arrest and, eventually, cell death. It has also been demonstrated that phen complexed with a metal has significantly higher activity than phen alone (36). For this reason, studies investigating phen as a potential cancer therapeutic often utilize phen-metal complexes (37, 38). If we assume that the inhibitory activity of 5-Nitro-1,10-phenanthroline is mechanistically similar to that of phen, the availability of metal ions could explain the differences in 5-Nitro-1,10-phenanthroline treatment outcomes between U2OS cells and primary keratinocytes or CIN612 cells. U2OS cells are grown in the presence of 10% fetal calf serum, while keratinocytes and CIN612 cells are grown in a defined serum-free medium. Serum contains high concentrations of Fe^2+^, Zn^2+^, and Cu^2+^—three metals that efficiently form complexes with phen. Therefore, a metal-5-Nitro-1,10-phenanthroline complex with high biological activity forms in U2OS cells but not in keratinocytes or CIN612 cells, leading to cell cycle arrest, possibly by inducing DNA intercalation-dependent damage. Alternatively, though less likely, the NSC4263 compound may not affect the cell cycle of epithelial-origin cells (keratinocytes and CIN612 cells), while mesenchymal-origin cells (U2OS) are sensitive to the compound.

Regardless of the precise mechanism of action of 5-Nitro-1,10-phenanthroline, we believe that its primary effect on HPV replication is indirect and occurs through cell cycle arrest. Furthermore, as previously mentioned, NSC4263 did not pass the REOS filter for druggability, making it an unlikely candidate for further drug development.

Another compound identified in the screen was 5,7-Dimethoxy-2-pyridin-3-ylchromen-4-one (NSC51349). Unlike the previously described NSC4263, this compound was effective only in suppressing the replication of cutaneous β-HPV types 5, 8, and 38, but not mucosal types 11, 18, 16, and 31. In addition, the compound directly affected viral replication rather than cell homeostasis, as it did not adversely impact the viability or cell cycle of U2OS cells or HPKs at concentrations below the IC90 (32 ± 1.6 and 10 ± 2 µM for U2OS and HPKs, respectively). Importantly, NSC51349 inhibited stable replication of the HPV5 genome in U2OS cells, which are known to support at least two different mechanisms of HPV replication (Fig. 5) (25, 26). One mechanism is E1-/E2-independent and likely involves recombination-dependent replication machinery of the host cell. The other mechanism depends on the E1 and E2 proteins, with viral replicons following a bidirectional mode of replication, similar to transient replication. We demonstrated that the NSC51349 compound specifically inhibits the E1- and/or E2-dependent stable replication of the HPV5 genome, leaving other types of replication unaffected (Fig. 5).

This compound has been shown to inhibit the nuclear peroxisome proliferator-activated receptor gamma (PPARγ), which acts as a ligand-dependent transcription factor with pro-apoptotic and anti-proliferative activities (39, 40). It has also been identified as an inhibitor of WEE1 kinase degradation in HeLa cells (41). WEE1 is a tyrosine kinase that regulates cell entry into the S phase and mitosis (42). An HTS has also identified the NSC51349 compound as an activator of the aryl hydrocarbon receptor, which is a ligand-dependent helix-loop-helix transcription factor (43). However, these activities of NSC51349 cannot account for its inhibition of HPV replication for the following reasons: (i) the compound specifically targets the replication of cutaneous β-HPVs, whereas inhibition of PPARγ and WEE1 should affect the replication of all HPV types and (ii) inhibition of these cellular proteins would be expected to impact either the cell cycle or viability, which was not observed. Interestingly, a recent *in silico* study identified the structurally similar to NSC51349 bioflavonoid apigenin and its derivatives as potential drug candidates for HPV-associated cervical cancer, capable of interacting with the HPV45 E7 oncoprotein and L1 major capsid protein (44). However, this computational investigation requires further validation *in vitro* and *in vivo*.

To gain insight into the mechanism of action of NSC51349, we performed a series of co-transfection experiments using combinations of replication-deficient HPV5 genomes and an E2 expression vector. The results of these experiments provide evidence that NSC51349 inhibits the transcriptional activity of the E2 protein, but not its replication-promoting activity. In other words, in the presence of NSC51349, E2 is still able to bind its cognate sites, form a complex with E1, and support bidirectional replication of the viral genome. However, NSC51349 specifically inhibits the ability of E2 to regulate transcription from the viral promoter. Therefore, the overall inhibitory effect of NSC51349 in the context of viral infection stems from the inability of viral genomes to induce the expression of viral early genes, including the *E6* and *E7* oncogenes, which is achieved through the positive autoregulatory loop of E2 activity.

The question of how exactly NSC51349 inhibits E2 transcriptional activity remains to be elucidated. Our molecular docking analysis predicted that the compound potentially binds between two peptide regions: one located in the C-terminal DNA-binding/dimerization domain and the other at the junction between the N-terminal TAD and the hinge region (Fig. 8). The residues predicted to interact with the inhibitor are highly conserved among beta HPVs, and the biological activity of the E2 protein is lost either through inhibitor binding or through mutations at these highly conserved residues. Since both mutations and inhibitor treatment result in the same effect, this strongly supports the conclusion that the identified residues participate in ligand binding.

The predicted binding site suggests at least three possible mechanisms for NSC51349-mediated inhibition. One possibility is that the altered affinity of the E2-NSC51349 complex to the E2-binding sites (BSs) involved in the transcriptional regulation of HPV5 plays a role. Four canonical and one noncanonical E2-binding sites with slightly different primary sequences have been mapped to the HPV5 URR (32). Although the differential requirements of these sites for HPV5 URR replication have been analyzed in detail, it is still unclear whether and to what extent different E2 BSs are involved in HPV5 transcriptional regulation. Analysis of mucosal HPVs suggests an E2 concentration-dependent model of viral transcriptional regulation, wherein E2 can mediate transcriptional repression or activation depending on the sites it binds to and their proximity to the transcription initiation site (45). Moreover, it has been shown for HPV11 that E2-binding affinity may vary depending on the flanking and spacer region sequences as well as the proximity of E2 BSs (46). Although E2 proteins of β-HPVs act solely as transcriptional activators, it is plausible to speculate that, similar to mucosal HPVs, cutaneous HPV E2 occupies different sites to mediate its transcription- and replication-related activities and may have different affinities for its BSs.

Another explanation for the inhibitory action of NSC51349 may be a reduced affinity of E2-NSC51349 for the cellular interactors involved in the regulation of viral transcription. In addition to directly regulating viral gene expression, E2 also recruits a range of host cell proteins to viral promoters (47). The N-terminal TAD of E2 interacts with at least 26 host cell proteins, many of which are involved in the transcriptional regulation of viral gene expression. One of the best-characterized E2-interacting proteins is the transcriptional and epigenetic regulator BRD4, a bromodomain-containing protein. It has been shown that the E2-BRD4 interaction is necessary for viral gene transcription, replication initiation, and genome segregation and maintenance (48). A set of small-molecule inhibitors developed to target the interaction of the phosphorylated BRD4 with E2 of HR α-HPVs has been shown to interfere with both the transcription- and replication-related activities of E2 (49).

Finally, the threonine residue T202, predicted to be involved in the interaction with NSC51349, and the nearby serine residue S206 have been identified as phospho-acceptor sites (31). The simulated E2 structure (Fig. 8A) suggests that these residues are located in an intrinsically disordered region of the protein, rich in proline-followed serine/threonine residues. Such linear motifs are often subjected to hierarchical multisite phosphorylation, mediated by proline-directed protein kinases (e.g., cyclin-dependent kinases), which rely on an initial phosphorylation event that primes subsequent modifications of adjacent residues (50). Although the functional significance of T202/S206 phosphorylation is unknown, it is possible that NSC51349 ligand binding prevents the attachment of phosphate groups to these residues, potentially leading to dysregulation of the E2 protein.

In summary, we identified a novel, passively cell-permeable small-molecule inhibitor that targets cancer-associated HPV5 replication by inhibiting E2-dependent transcription. Our study opens the possibility of optimizing the structure of this lead compound through structure-based drug design approaches to enhance its efficacy, thus leading to the development of novel antiviral drugs.

## MATERIALS AND METHODS

### Plasmids and expression constructs

HPV5, HPV5-Nluc, HPV5-E1fs, HPV5-E2fs, HPV5-E1fs-E2fs, HPV11, HPV11-Nluc, HPV18, HPV18-Nluc, HPV38, HPV16, and *M*. *fascicularis* PV genomes and their parental plasmids were described previously (19, 29, 31, 33, 51, 52). The HPV5-E2fs-Nluc genome was generated by inserting Nluc and foot-and-mouth disease virus 2A self-processed peptide-encoding sequences into the HPV5-E2fs genome, as described previously (19). All viral genomes were generated from the respective pMC.BESBX parental plasmids using minicircle technology (53). The expression vectors coding for the HPV5 E2-Flag, HPV5 E1-HA, and HPV18 E1-HA have been previously described (24, 29, 54). To create expression constructs encoding HPV5 E2 proteins with alanine substitutions for amino acid residues predicted to interact with the inhibitor, synthetic DNA fragments containing the required mutations (purchased from Twist Bioscience) were cloned into the codon-optimized E2 sequence. The resulting constructs were verified by DNA sequencing.

### Chemicals

Diversity Set VI library and the additional compounds NSC4263 and NSC51349 were obtained from the Drug Synthesis and Chemistry Branch, Developmental Therapeutics Program, Division of Cancer Treatment and Diagnosis, National Cancer Institute. An additional amount of 5,7-dimethoxy-2-pyridin-3-ylchromen-4-one (purity ≥98%) was purchased from Celestial Specialty Chemicals (Latvia). The chemicals were dissolved in DMSO (Sigma-Aldrich).

### Cell culture

Human osteosarcoma cell line U2OS (ATCC no HTB-96) was propagated in normal growth medium (NGM) containing Iscove modified Dulbecco medium (Corning), 10% fetal calf serum, and 1% penicillin-streptomycin (Sigma-Aldrich). The cells were transfected by electroporation using a Gene Pulser XCell system (Bio-Rad Laboratories) as described previously (19). The HPV5 +stable cell line containing the extrachromosomal HPV5 genome was described previously as clone 15 (20, 24).

HPV31b-positive CIN612E cells and HPKs (kind gift from Dr. Frank Stubenrauch and Promocell or CELLnTEC, respectively) were propagated in Defined Keratinocyte-SFM Medium (Gibco, cat. no. 17005042) supplemented with penicillin-streptomycin as previously described (19). Keratinocytes were passaged every 3–4 days by detachment with 0.025% Trypsin-EDTA for 2 minutes at 37°C, followed by immediate transfer into fresh medium, centrifugation at 1400 rpm for 3 minutes, and re-plating in new dishes with fresh medium. On the 2nd day after seeding on 96-well plates, the HPKs were transfected using Lipofectamine LTX supplemented with Plus reagent (Invitrogen). Approximately 80 ng of the viral genomes was added to each well. Differentiation of the HPKs was induced with 1.5 mM CaCl_2_ in keratinocyte medium containing threefold less supplements than used to culture undifferentiated cells. Cell cycle analysis using propidium iodide was conducted as previously described (55). Cellular viability was analyzed using the Cell Meter Colorimetric WST-8 Cell Quantification Kit (VWR) or the CellTiter 96 AQueous Nonradioactive Cell Proliferation Assay Kit (MTS) (Promega).

### HTS for identification of HPV5-Nluc inhibitors

HTS was conducted as shown in Fig. 1A and B. The scheme was drawn using BioRender software. Approximately 10^6^ U2OS cells were transfected with 1.5 µg of the HPV5-Nluc genome and 5 µg of salmon sperm DNA as a carrier. The cells were seeded in black Nunc MicroWell 96-well plates with a clear optical bottom (Thermo Scientific). The next day, the Diversity set VI compounds were added to the media at a concentration of 10 µM, and the cells were incubated for an additional 2 days. Nluc activity was measured using the Nano-Glo Live Cell Assay System (Promega) directly in the cell culture microplates and normalized to the number of viable cells, which was measured using the CellTiter 96 AQueous Nonradioactive Cell Proliferation Assay Kit (MTS) (Promega). For measurement, the normal growth medium was replaced with 100 µL/well of pure IMDM containing the Nluc and MTS substrates simultaneously, and Nluc activity was measured immediately. Next, the plates were incubated at 37°C for 30 min, and absorbance was measured at 490 nm. The second round of HTS was conducted with selected chemicals at concentrations of 10 and 20 µM, with incubation times of 2, 3, and 4 days post-transfection. Finally, the inhibitory effects of the selected chemicals were confirmed using Southern blotting (SB).

### RNA interference

HPV5- or HPV5-E1HA-Nluc-E2Flag-positive U2OS-derived cells were transfected with 40 nM scramble, E1- or E2-specific siRNAs using electroporation. The following siRNAs were used: *E1* siRNA1 GUGGGAUAGGUGCAAUGUCAU, *E1* siRNA2 GGGCACUGGUCAGAUAUAGUA, *E2* siRNA1, GAGCCAUGGACUCUAGUUGAU, *E2* siRNA2 GAGAAAGGUGUUACAAGGCUU, and Neg. siRNA UAGCGACUAAACACAUCAA. Cells were incubated for up to 5 days, and LMW DNA was isolated and analyzed using SB. Alternatively, whole-cell extracts were prepared for immunoblotting of GAPDH and immunoprecipitation of the E1 and E2 proteins.

### Immunoprecipitation and Western blotting

Approximately 10^6^ cells were used for each immunoprecipitation of the endogenous HA-tagged E1 and Flag-tagged E2 proteins. Cells were lysed in RIPA buffer (50 mM Tris pH 7.5, 150 mM NaCl, 2 mM EDTA, 0.1% SDS, and 0.1% TRITON-X100) supplemented with protease inhibitor cocktail (PIC, Roche). Lysates were incubated with r-a-HA (19) or polyclonal rabbit-anti-E2 antibodies (31) (Labas Ltd., 2 µg per sample) and Dynabeads Protein G (Invitrogen, 4 µL per sample) at 4°C and slow rotation overnight. Immuno-complexes were washed 3 times with 1 mL of RIPA buffer, lysed in Laemmli sample buffer. To analyze the overexpressed Flag-tagged WT and mutant E2 proteins, the cells were lysed directly on the culture plates using Laemmli lysis buffer. All samples were denatured at 100°C for 5 min and subjected to SDS-PAGE analysis. The following antibodies were used for immunoblotting: HA-3F10-HRP (Roche), rabbit-anti-E2, Flag-M2-HRP (Sigma-Aldrich) in dilution 1:2500, and GAPDH-HRP (Invitrogen) in dilution 1:8,000.

### Southern blot analysis

An analysis of the linearized HPV genomes of types 11, 18, and 5 was performed using 5 µg of total DNA digested with the following FastDigest restriction enzymes: HindIII for HPV11, BglI for HPV18, and SacI for HPV5 and its derivatives. The HPV8, HPV38, and HPV16 genomes were detected using 40 µg of Hirt extract and the restriction endonucleases BglII, BglI, and HindIII, respectively, for genome linearization. In addition, DpnI was used to cut the bacterially methylated input DNA. The linearized HPV31b genome was analyzed using 2 µg of total DNA and the EcoRI restriction enzyme. To analyze the pattern of the uncut HPV31b genome, 15 µg of LMW DNA was treated with the HPV31 noncutter restriction enzyme BamHI. To analyze the pattern of the uncut HPV5 genome, 20 µg of LMW DNA was treated with NdeI or NheI. Mf PV genomes were analyzed as described previously (33) using BamHI and SacI as noncutters for the Mf PV1 and Mf PV5/8, respectively. All restriction enzymes were purchased from Thermo Fisher Scientific, and restriction reactions were performed in FastDigest Green buffer at 37°C for at least 5 h. The isolation of total and LMW DNA, DNA separation, transfer, and hybridization were performed as described previously (20, 25). SB signals corresponding to the replicated HPV genomes were quantified using ImageQuant software.

### RNA isolation and qRT-PCR

Total RNA was isolated using the Direct-Zol RNA Miniprep Plus kit (Zymo Research). Approximately 8 µg of total RNA was treated with 2 µL of Turbo DNase (Invitrogen) for 3 hours at 37°C. To inactivate the DNase, the samples were incubated at 75°C for 10 minutes in the presence of 15 mM EDTA. The total RNA was then precipitated with 7.5 M LiCl supplemented with 50 mM EDTA and washed with 70% ethanol. Complementary DNA (cDNA) was synthesized from 3 µg of total RNA using the RevertAid cDNA synthesis kit (Thermo Fisher Scientific). The expression levels of housekeeping genes *GAPDH* and *ACTB*, HPV5 transcripts, cell cycle-related *Cyclins A*, *B*, *D*, and *E*, as well as differentiation markers *K10, K14*, *INV*, and *LOR*, were measured in triplicate by quantitative PCR using 5× HOT FIREPol Blend Master Mix with 12.5 mM MgCl_2_ (Solis Biodyne). The ΔΔCt method was used to calculate the relative fold changes in gene expression levels. The primers used in the present study were described previously (19, 31, 56).

### Luciferase assay

Cells were grown in 96-well plates, washed twice with PBS, and lysed in Cell Culture Lysis Solution (Promega) using 50 µL of buffer per well. Nluc activity was measured using 10 µL of lysate and 25 µL of a solution containing furimazine substrate diluted 1:500 in a buffer consisting of 50 mM MES (pH 6.0), 0.5 mM EDTA, 75 mM KCl, 1 mM DTT, and 18 mM thiourea (57). AP activity was measured using 10 µL of lysate and 25 µL of CSPD substrate with Sapphire-II Enhancer (Invitrogen). Total protein concentrations were determined using the BCA Protein Assay Kit (Pierce Biotechnology). AP activity and total protein concentrations were used to normalize the Nluc activity values.

### E2 protein structure prediction and minimization

The 3D structure of the full-length E2 protein was predicted using AlphaFold with MMseqs2 MSA and subsequently processed using the Schrödinger Protein Preparation Wizard (58). The prepared protein structure was then minimized using a 30 ns MD simulation via the Desmond program package from Schrödinger LLC (34). The MD-optimized structure was used for further modeling. The MD simulations were performed as previously described (59). The trajectory analysis was conducted using the Simulation Interaction Diagram tool, implemented in the Desmond molecular dynamics package. The quality of the MD simulations was assessed using the Simulation Quality Analysis tool, also part of the Desmond package. The root mean square deviation (RMSD) of the Cα atom positions over time was monitored to assess the stability of the predicted E2 structure. The protein structure corresponding to the 50 ns time point of the MD simulation was saved in a separate PDB file and used for further modelling.

### Molecular docking

The NSC51349 structure, downloaded from PubChem in .mol2 format, and the MD-minimized E2 protein structure were prepared for docking using AMDock software at pH 7.4 (60, 61). The potential binding site of the E2 protein was identified using the AutoLigand tool (35). For the docking procedure, the binding site was enclosed in a grid box with a spacing of 1 Å, and default docking parameters were used (1 CPU, exhaustiveness of 8, and 9 output poses). Further analysis of the docking results was performed using PyMol software (62).

## Data Availability

All relevant data are included within the article and its supplemental material.

## SUPPLEMENTAL MATERIAL

Fig. S1 jvi.00918-25-s0001.pdf U2OS cells were transfected with the WT HPV5 genome or HPV5 encoding the Nluc gene and scrambled, E1, or E2 siRNAs.

Fig. S2 jvi.00918-25-s0002.pdf Additional cell transfection results and Ramachandran plot.

Fig. S3 jvi.00918-25-s0003.pdf Uncropped images.

Supplemental legends jvi.00918-25-s0004.docx Legends for Fig. S1 and S2.

Table S1 jvi.00918-25-s0005.xlsx Results of initial HTS of the NCI Diversity Set VI library of small molecule compounds in U2OS cells.

ASM does not own the copyrights to Supplemental Material that may be linked to, or accessed through, an article. The authors have granted ASM a non-exclusive, world-wide license to publish the Supplemental Material files. Please contact the corresponding author directly for reuse.
