# Supplementary figures and images for: Targeting papillomavirus infections: high-throughput screening reveals an effective inhibitor of cutaneous β-HPV types
Source: J Virol. 2025 Jul 8;99(8):e00918-25. doi: 10.1128/jvi.00918-25 (PMC12363231; doi:10.1128/jvi.00918-25)

A

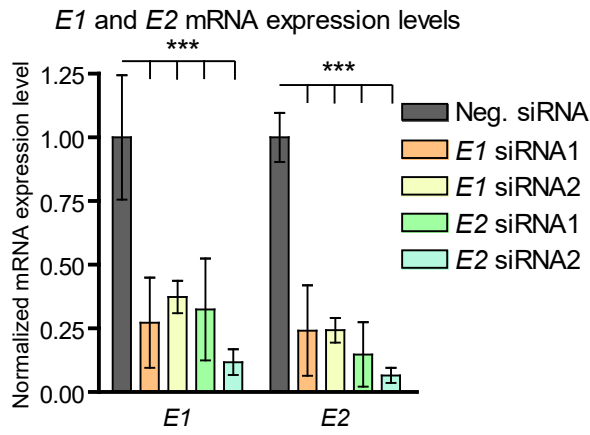

B

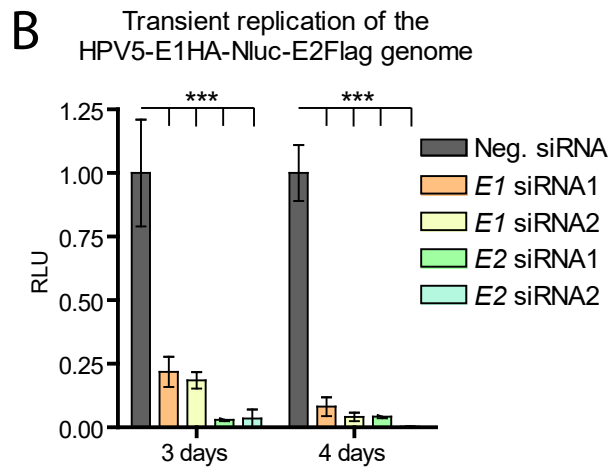

C

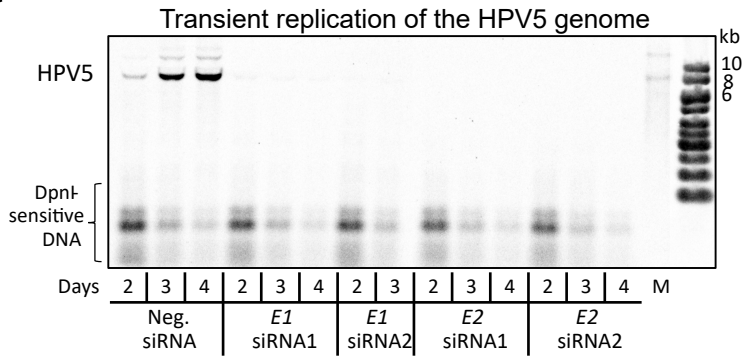

D

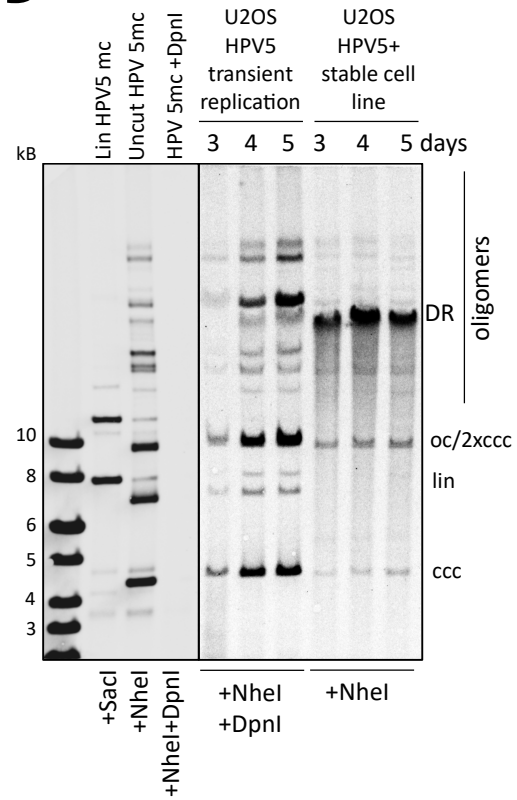

E

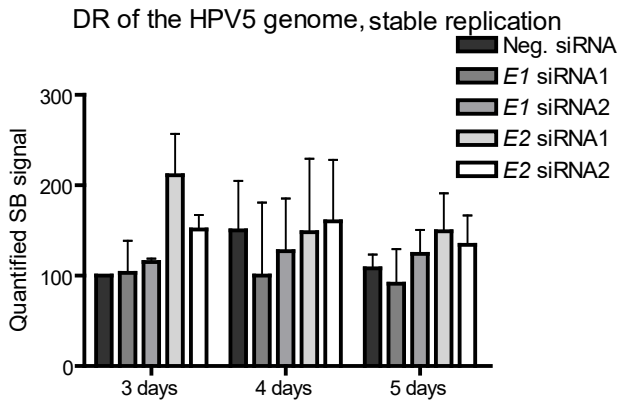

**Linearized HPV5 genome, stable replication**

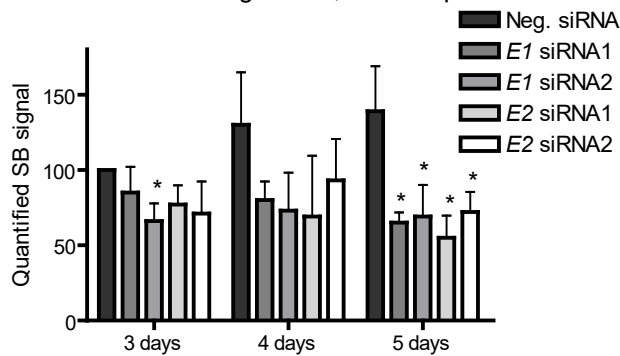

Supplement: Fig. S1 — U2OS cells were transfected with the WT HPV5 genome or HPV5 encoding the Nluc gene and scrambled, E1, or E2 siRNAs. [file jvi.00918-25-s0001.pdf]

A

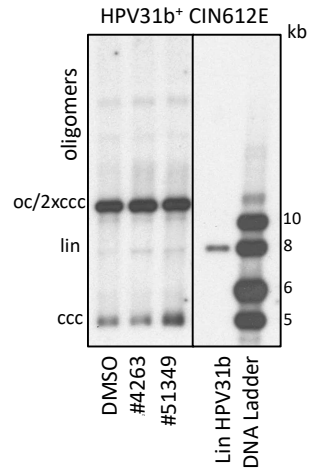

B

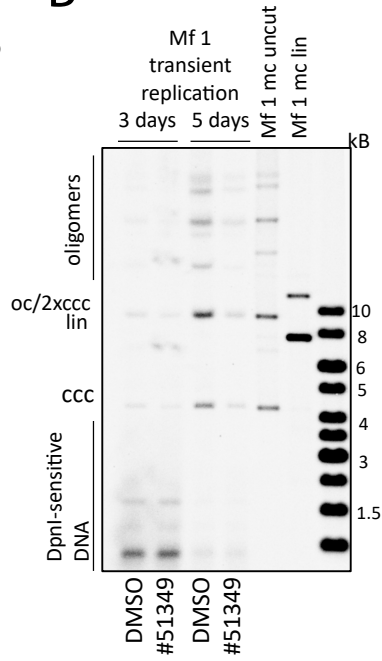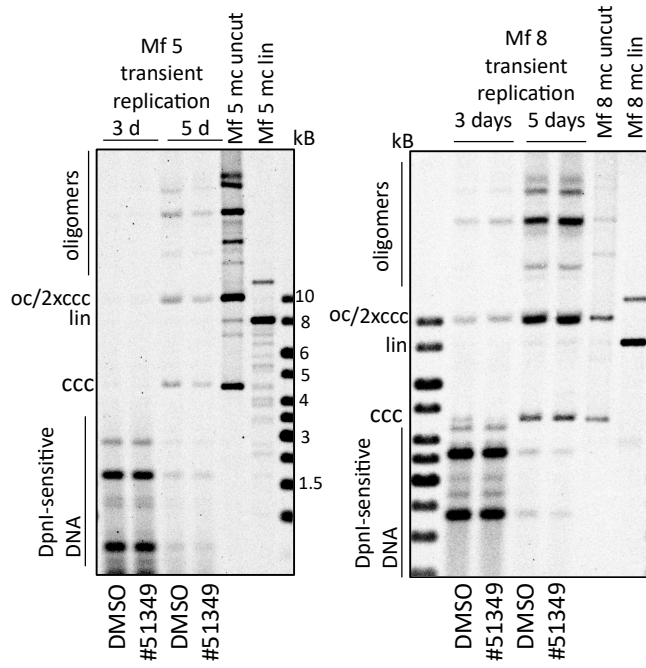

C

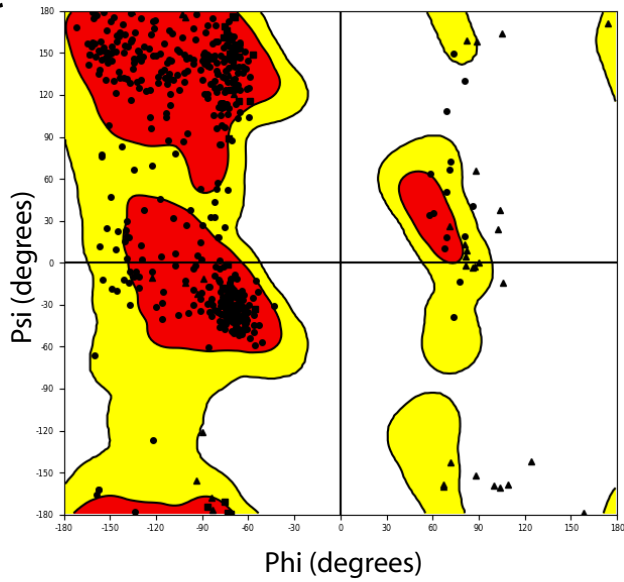

D

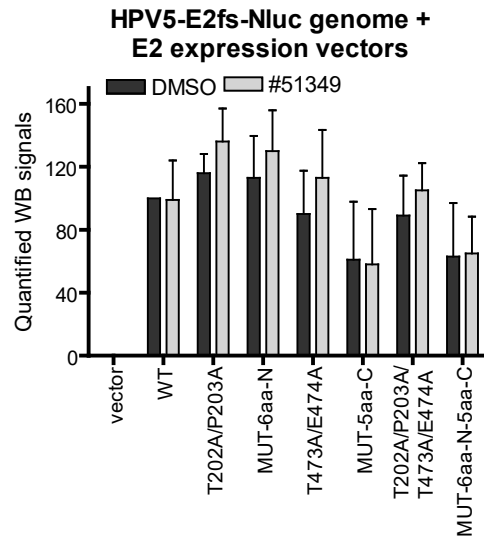

Supplement: Fig. S2 — Additional cell transfection results and Ramachandran plot. [file jvi.00918-25-s0002.pdf]

Figure 2B

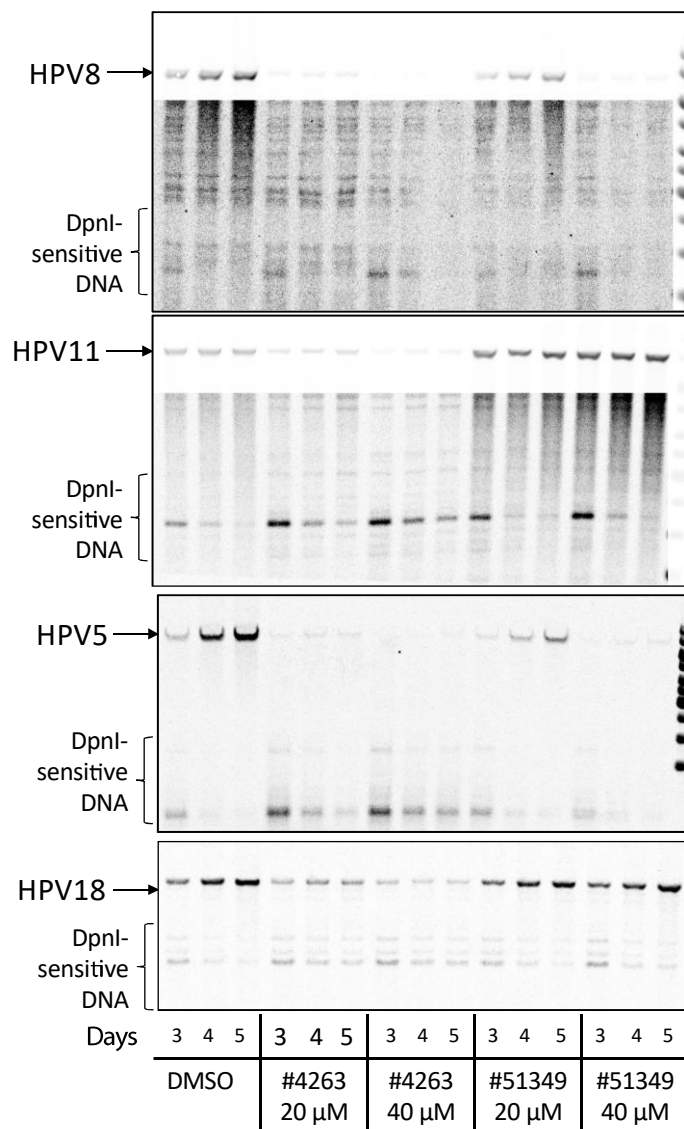

Figure 2C

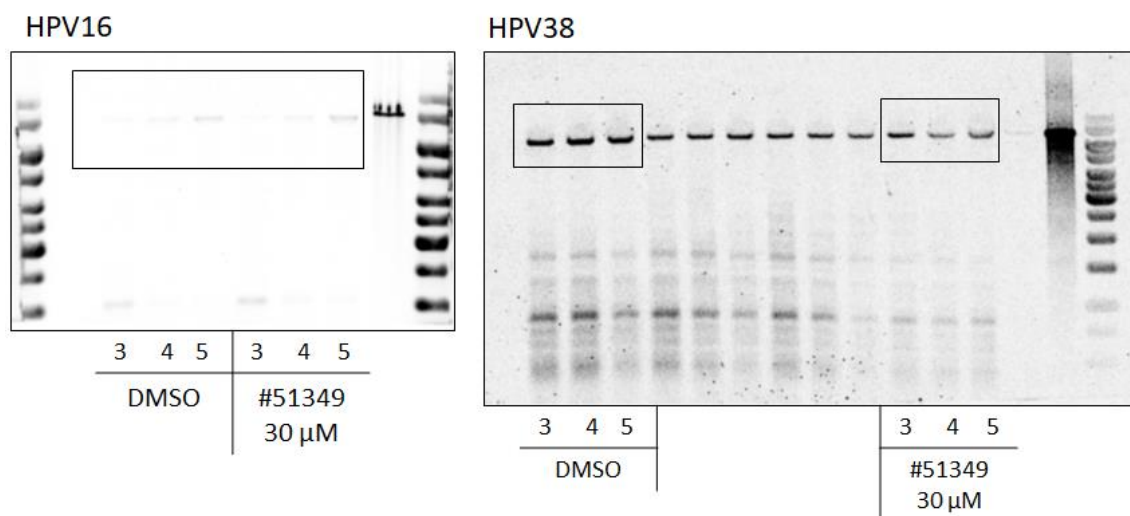

Figure 4D

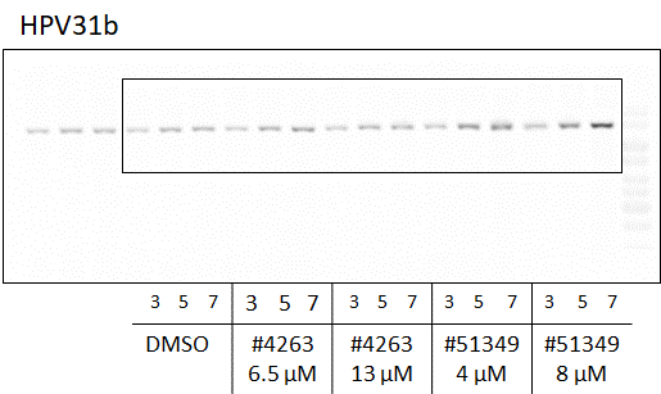

Figure 5A

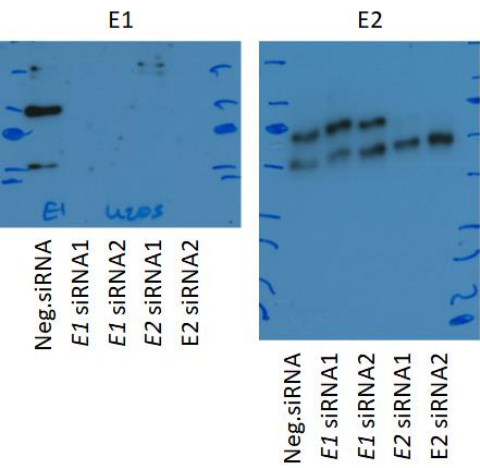

Figure 6D

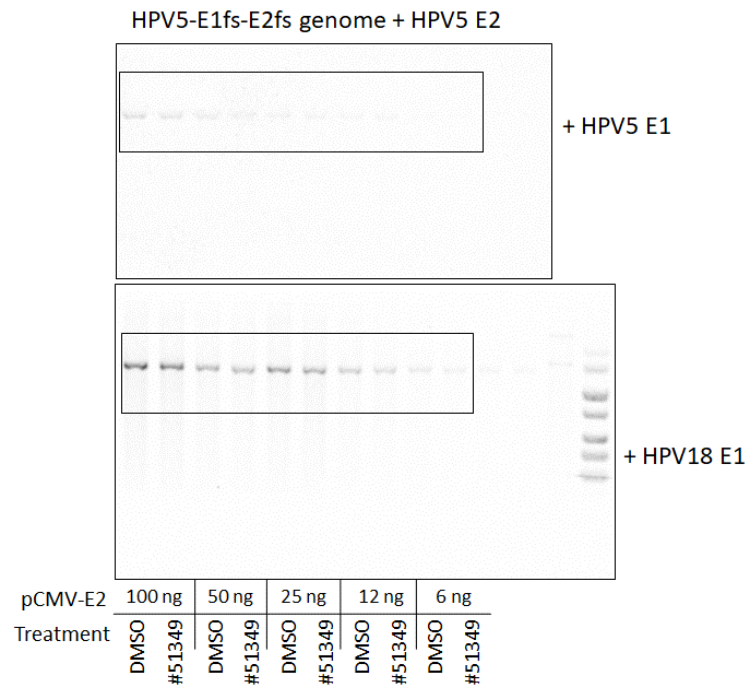

Figure 8D

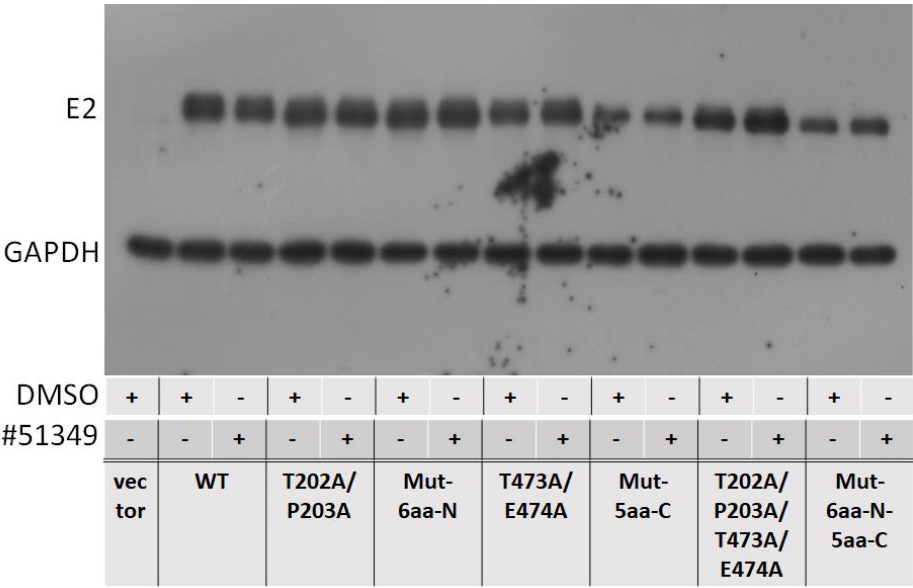

Supplement: Fig. S3 — Uncropped images. [file jvi.00918-25-s0003.pdf]
