# Supplementary material for: Targeting papillomavirus infections: high-throughput screening reveals an effective inhibitor of cutaneous β-HPV types
Source: J Virol. 2025 Jul 8;99(8):e00918-25. doi: 10.1128/jvi.00918-25 (PMC12363231; doi:10.1128/jvi.00918-25)
Supplement: Supplemental legends — Legends for Fig. S1 and S2. [file jvi.00918-25-s0004.docx]

**Supplementary Figure Legends**

**Supplementary Fig S1. A, B, C:** U2OS cells were transfected with the either WT HPV5 genome or HPV5 encoding the Nluc gene and scrambled (Neg.), *E1*, or *E2* siRNAs. The level of viral gene expression was measured using qRT-PCR three days post-transfection, normalized to *GAPDH* expression levels, and set to 1 in the control samples transfected with Neg. siRNA (A). Viral genome copy numbers were estimated using an Nluc assay in U2OS cells three and four days post-transfection. Nluc activity, normalized to AP values, was set to 1 in the control samples (B). Data from other samples were calculated relative to the control. Data are presented as the mean of at least three independent experiments ± SD; p < 0.001. Total DNA was isolated at the indicated time points and treated with DpnI to digest the input viral DNA and SacI to linearize the HPV5 genome. DNA was transferred to a membrane, and hybridized with the radioactively labelled HPV5-specific probe (C). **D**. U2OS cells were transfected with the HPV5 genome and incubated for 3, 4, and 5 days (HPV5 transient replication). U2OS-derived cells bearing the HPV5 genome (HPV5+ stable cell line) were propagated for 3, 4, and 5 days. LMW DNA was isolated and treated with the HPV5-noncutting restriction enzyme NheI, followed by SB analysis. HPV5 transient replication samples were treated also with DpnI to remove the input DNA purified from bacteria. The patterns of the linearized (lin), uncut, and DpnI- and NheI-digested HPV5 minicircle (mc) used for the transient transfection of U2OS cells and the generation of the stable cell line are shown in the left panel; ccc, covalently closed circular DNA; lin, linear DNA; oc, open circular DNA. **E**. U2OS-derived cells bearing the HPV5 genome were treated with the indicated siRNAs and incubated up to 5 days. LMW DNA was isolated and treated with either the SacI restriction endonuclease to linearize the HPV5 genome or the HPV5-noncutting restriction enzyme NdeI, followed by SB analysis. SB signals shown in Figure 5B for the dominant replicon (DR) and the linearized HPV5 genome were quantified and set to 100% in the sample transfected with Neg. siRNA and incubated for 3 days. Data from other samples are presented relative to this control (p < 0.05, n = 3).

**Supplementary Figure S2 A.** CIN612E keratinocytes were treated with 8 μM NSC4263 and 13 μM NSC51349 for 5 days. LMW DNA was treated with HPV31 noncutter restriction enzyme BamHI and subjected to SB analysis. **B.** U2OS cells were transfected with the M. fascicularis genomes type 1, 5, and 8 and treated with either NSC51349 or DMSO. LMW DNA was isolated, treated with DpnI and noncutter restriction enzymes BamHI for Mf1, and SacI for Mf5 and Mf8, and analysed using SB (with days post-transfection are indicated), mc – a minicircle DNA used for transfection. A and B: ccc, covalently closed circular DNA; lin, linear DNA; oc, open circular DNA. **C**. Ramachandran plot of the MD minimized E2 structure. The red and yellow regions represent the favoured and allowed regions, respectively. Glycine amino acid residues are shown as triangles, and other amino acid residues are shown as dots. The plot was generated by Maestro Schrödinger. **D**. U2OS cells were transfected with the HPV5-E2fs-Nluc genome and expression constructs encoding the indicated HPV5 Flag-tagged E2 proteins or an empty vector. Next day, cells were treated with DMSO or 20 µM NSC51349 for 24 h. Expression levels of the E2 proteins were analyzed using immunoblotting. The signals obtained from the control samples transfected with WT E2 and treated with DMSO were set to 100%, and data from the other samples are presented as a percentage of the control +/- SD.

**S3 Data** Uncropped images
